# Forecasting the 2013–2014 Influenza Season Using Wikipedia

**DOI:** 10.1371/journal.pcbi.1004239

**Published:** 2015-05-14

**Authors:** Kyle S. Hickmann, Geoffrey Fairchild, Reid Priedhorsky, Nicholas Generous, James M. Hyman, Alina Deshpande, Sara Y. Del Valle

**Affiliations:** 1 Theoretical Division Los Alamos National Laboratory, Los Alamos, New Mexico, United States of America; 2 Defense Systems Analysis Division Los Alamos National Laboratory, Los Alamos, New Mexico, United States of America; 3 High Performance Computing Division Los Alamos National Laboratory, Los Alamos, New Mexico, United States of America; 4 Department of Mathematics, Tulane University, New Orleans, Louisiana, United States of America; Pennsylvania State University, UNITED STATES

## Abstract

Infectious diseases are one of the leading causes of morbidity and mortality around the world; thus, forecasting their impact is crucial for planning an effective response strategy. According to the Centers for Disease Control and Prevention (CDC), seasonal influenza affects 5% to 20% of the U.S. population and causes major economic impacts resulting from hospitalization and absenteeism. Understanding influenza dynamics and forecasting its impact is fundamental for developing prevention and mitigation strategies. We combine modern data assimilation methods with Wikipedia access logs and CDC influenza-like illness (ILI) reports to create a weekly forecast for seasonal influenza. The methods are applied to the 2013-2014 influenza season but are sufficiently general to forecast any disease outbreak, given incidence or case count data. We adjust the initialization and parametrization of a disease model and show that this allows us to determine systematic model bias. In addition, we provide a way to determine where the model diverges from observation and evaluate forecast accuracy. Wikipedia article access logs are shown to be highly correlated with historical ILI records and allow for accurate prediction of ILI data several weeks before it becomes available. The results show that prior to the peak of the flu season, our forecasting method produced 50% and 95% credible intervals for the 2013-2014 ILI observations that contained the actual observations for most weeks in the forecast. However, since our model does not account for re-infection or multiple strains of influenza, the tail of the epidemic is not predicted well after the peak of flu season has passed.

## Author Summary

We use modern methods for injecting current data into epidemiological models in order to offer a probabilistic evaluation of the future influenza state in the U.S. population. This type of disease forecasting is still in its infancy, but as these methods become more developed it will allow for increasingly robust control measures to react to and prevent large disease outbreaks. While weather forecasting has steadily improved over the last half century and become ubiquitous in modern life, there is surprisingly little work on infectious disease forecasting. Although there has been a great deal of work in modeling disease dynamics, these have seldom been used to generate a probabilistic description of expected future dynamics, given current public health data. Moreover, the mechanism to update expected disease outcomes as new data becomes available is just beginning to receive attention from the public health community. Using CDC influenza-like illness reports and digital monitoring sources, such as observations of Wikipedia article access logs, we are now at a point where forecasting for the influenza season can begin to offer useful information for disease monitoring and mitigation.

## Introduction

Despite preventive efforts and educational activities for seasonal influenza in the United States (U.S.), on average 5%–20% of the population gets influenza [1], more than 200,000 people [1] are hospitalized from seasonal influenza complications, and 3,000–49,000 people die each year [2]. The result is a significant public health and economic burden for the U.S. population [3–5].

The Centers for Disease Control and Prevention (CDC) monitor influenza burden by collecting information from volunteer public health departments at the state and local level [6–9]. Data are then used for planning and mitigation activities based on what is believed to be the current state of influenza throughout the U.S. [6, 7, 10]. These rough estimates could possibly lead to significant over- or under-preparation for any given flu season.

In November of 2013, the CDC launched the *Predict the Influenza Season Challenge* competition to evaluate the growing capabilities in disease forecasting models that use digital surveillance data [11]. The competition asked entrants to forecast the timing, peak, and disease incidence of the 2013–2014 influenza season using Twitter or other Internet data to supplement ILI weekly reported data. The work described in this paper was conducted as an entry for this competition. The CDC challenge concluded on March 27^th^, 2014. Though individual entries’ scores were not announced, the winner was. Officials conducting the contest found each entry informative as to the capabilities of disease modeling to inform public policy but the general consensus was that the current state of influenza forecasting still has too much uncertainty to base public health policy on. Details of the contest will be announced in a forthcoming paper by the CDC [12]. Based on our entry to this contest, we present a novel method to provide a probabilistic forecast of the influenza season based on a mathematical model for seasonal influenza dynamics and historical U.S. influenza observations. The forecast is dynamically adjusted using a statistical filter as current influenza data is observed.

Reliable forecasts of influenza dynamics in the U.S. cannot be obtained without consistently updated public health observations pertaining to flu [13]. It is necessary to have a historical record of these observations in order to asses the relation between the forecasting model and the data source. Our primary data source, ILINet, is the CDC’s outpatient *influenza-like illness* (ILI) surveillance system [6, 14]. ILINet data represent the collection of outpatient data from over 3,000 hospitals and doctors’ offices across the U.S. Each week, these locations report the total number of patient visits and the number of those visits that were seen for ILI, defined as fever (temperature ≥ 100°F) and a cough or sore throat without a known cause other than influenza. Since 2003, these data have been collected weekly, year-round. ILINet data make up one portion of the CDC’s complete influenza surveillance efforts. For example, U.S. influenza-related mortality and virological strain data are also collected, but they are not used in this study. For a complete overview of the CDC’s influenza surveillance programs we refer the reader to [6, 7, 14].

Due to the ILINet dataset’s use by the CDC and other public health agencies in the U.S. together with ILINet’s availability and archived collection of historical influenza data, we develop our influenza forecast to predict ILINet. However, ILI represents a limited syndromic observation of people who seek medical attention each week [7, 10]. Furthermore, the distribution and specific reporting practices of the approximately 3,000 healthcare providers involved in ILINet, the lack of care-seeking for many individuals infected with influenza, and the possibility of ILI symptoms without infection from the influenza virus complicates the understanding of the relation between a reported level of ILI and actual U.S. influenza prevalence. An additional drawback is related to the bureaucratic hierarchy of the ILI system; there is a 1–2 week lag present in data availability.

We used Wikipedia article access logs to supplement the ILINet data and broaden the range and depth of information. The addition of new data sources that estimate influenza incidence can increase the robustness of the ILI data stream [15–19]. Wikipedia access logs for articles highly correlated with influenza prevalence, as measured by ILI, improve our knowledge of the current influenza incidence in the U.S. The rationale for using the Wikipedia access logs was thoroughly explored in reference [15].

Influenza forecasting must provide two things in order to inform public health policy: 1) the expected future influenza dynamics and 2) the likelihood of observing dynamics deviating from this expectation. These two properties are informed by both the inherent model dynamics and current observations of flu. Fortunately for epidemiologists, the methodology for generating probabilistic forecasts with a deterministic mathematical model based on observed data has been well developed in climatology, meteorology, and oceanography [20–22]. We demonstrate how the *ensemble Kalman smoother*, can be used to iteratively update a distribution of the influenza model’s initial conditions and parameterizations. An advantage of our technique is that it retains information about when the model dynamics systematically diverge from the dynamics of observations. The systematic model divergence from observed influenza dynamics will be used as a basis for future research in model discrepancy to improve forecasts.

The capability for real-time forecasting of events, such as influenza dynamics, with quantified uncertainty, has been crucial for major advances across the spectrum of science [20–22]. However, this capability is still in its infancy in the field of public health. For more complete literature reviews on the field, we refer the reader to [23, 24]. Briefly, we present the literature on epidemic forecasting influencing this work, all of which rely on a Bayesian viewpoint to adjust an underlying disease model given incoming observations [25]. First, disease forecasting methods that use data to parameterize an underlying causal model of disease can use either sequential Monte Carlo type methods [14, 26–31] or ensemble methods [32–35]. Some work has been done on comparing the two methods [36, 37]. There are also several works of a more statistical nature [38–43], one that relies on a pure Kalman filter [44], and one that uses variational assimilation methods [45]. Of these works, the majority tune a differential equation-based compartmental disease model [26, 28–30, 32, 33, 36, 37, 45]. However, some forecasts have been formed using agent based simulations [27, 31, 41] or spatial models [34, 35].

Each of these examples rely on defining a prior distribution for the parameterization and initialization of the underlying model. However, the methods to arrive at this prior are usually *ad hoc* and based on beliefs about the ranges for the parameters. It is therefore difficult to see how these methods may be applied to a general disease model given historic observations in the presence of model error. Our method for defining a prior parameterization/initialization can be generalized to any dataset pertaining to disease spread and disease model.

After outlining the general forecast methodology, we describe the details of our data sources and model, the technique used to estimate a *prior* forecast, our data assimilation technique, and our measure of forecast accuracy. We then present an application of our methods to forecasting the 2013–2014 influenza season in the results section, and conclude with a summary of our approach and suggestions for future improvements.

## Methods

### Wikipedia data

We identify correlations between CDC ILI data and Wikipedia access log data to improve our forecasts. There is a time delay in reporting of ILINet data. Wikipedia data, on the other hand, is available almost immediately and has the potential to provide information about the current state of influenza.

As mentioned above, there is a 1–2 week lag between a patient seeing a doctor and the case appearing in the ILI database. Therefore, there is a need for the use of digital surveillance data available in near real-time that can complement ILINet data. We turn to publicly available Wikipedia access log data to achieve this.

Wikipedia provides summary article access logs to anyone who wishes to use them. These summaries contain, for each hour from December 9, 2007 to present (and updated in real-time), a compressed text file listing the number of requests served for every article in every language, for articles with at least one request. Using the MapReduce programming paradigm [46], we aggregate these hourly requests into weekly access counts and normalize the total number of accesses per article using the total requests for all articles across the entire English Wikipedia in each week. Wikipedia access logs have been studied extensively in [15, 16], and we refer the reader to these sources for thorough analyses of the data. In short, it was shown that for a range of infectious diseases across many countries, some articles have access rates that are highly correlated with public health infectious disease records. Simple statistical models trained using only these article access rates were capable of nowcasting and even forecasting public health data, achieving *r* ^2^ ≥ 0.9 in certain cases.

Five articles from the English language edition of Wikipedia were selected for estimation of present national ILI using the methods outlined in [15]. These articles were *Human Flu*, *Influenza*, *Influenza A virus*, *Influenza B virus*, and *Oseltamivir*. To select these articles, we used the simple article selection procedure described in [15]: we first gathered access log time series for relevant articles linked to from the main influenza Wikipedia article [47], including the main influenza article itself. This totaled approximately 50 articles. The correlation between each of the 50 article access log time series and U.S. ILI data was computed. It was found that access log time series from the five articles mentioned above were much more highly correlated with the ILI data than the remaining articles, so it was decided that only these five articles would be used. It is important to note that only examining this restricted set of 50 Wikipedia access logs will inevitably leave the potential for the existence of an un-investigated article that is highly correlated with ILI data. However, barring an exhaustive search of Wikipedia articles focusing on the articles linked from the main English influenza article seemed like a reasonable choice.

The weekly article request data for each article can be written as the independent variables *x*_1_, *x*_2_, …, *x*_5_. Current ILI data are estimated using a linear regression from these variables. We combine the article request data with the previous week’s ILI data, which we’ll denote by *ILI*_−1_, and a constant offset term. This forms our regression vector *X* = (1, *x*_1_, *x*_2_, …, *x*_5_, *ILI*_−1_). Our linear model used to estimate the current week’s ILI data is then given by

$$\begin{array}{c} ILI_{0}=b\cdot X=b_{0}+b_{1}x_{1}+b_{2}x_{2}+\cdots +b_{5}x_{5}+b_{6}ILI_{-1}. \end{array}$$ (1)

**Fig 1:**
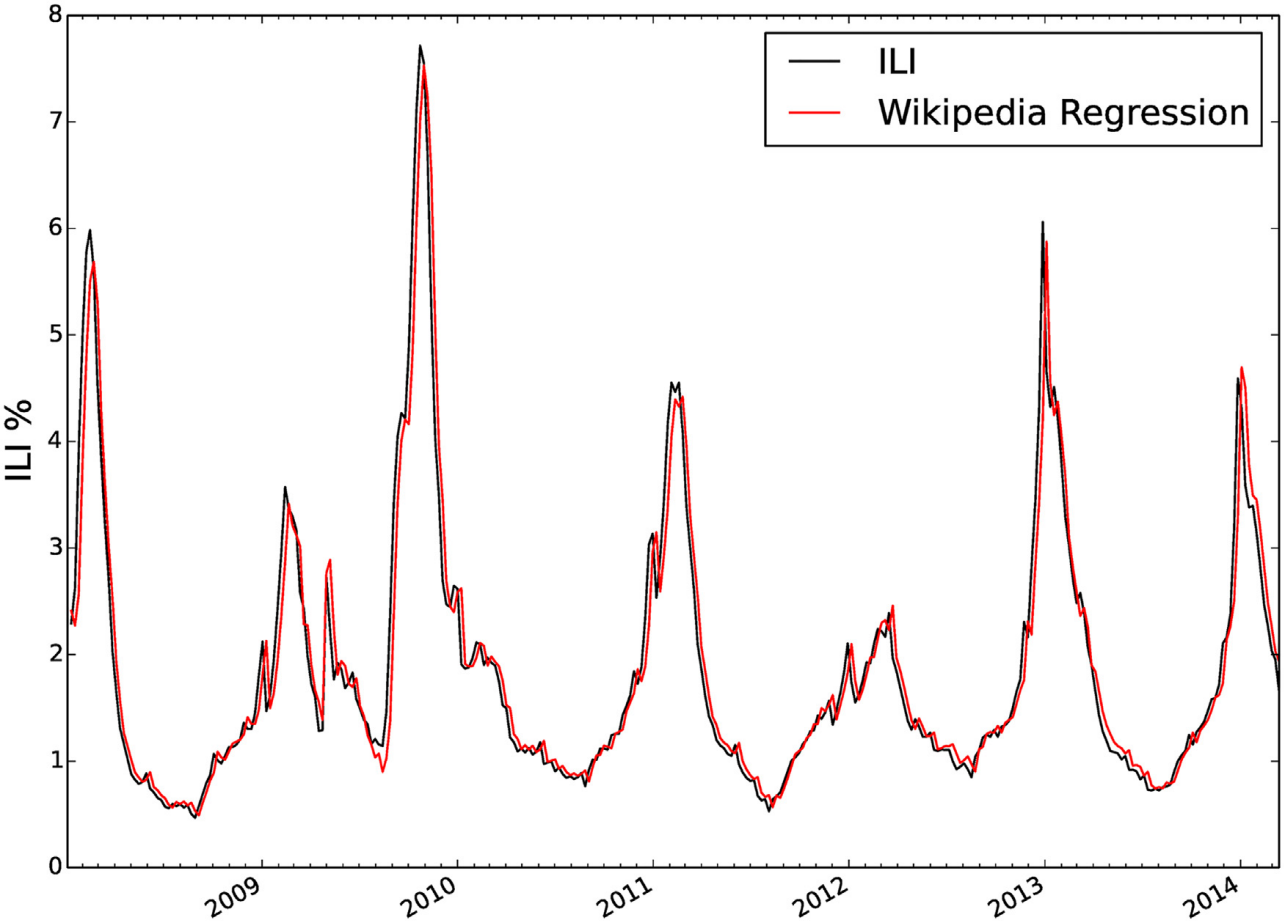
Regression of Wikipedia access logs to ILI data. Here we show the linear regression of one weeks prior ILI observation, a constant term, and the access logs of five Wikipedia articles related to influenza to the current ILI observation. The regression is highly correlated to the true ILI outcome. However, due to the simple form of this linear regression it is difficult to quantify which of the six regressors used was most influential in predicting ILI.

The regression coefficients *b* = (*b*_0_, *b*_1_, *b*_2_, …, *b*_6_) were then determined from historical ILI data and Wikipedia data. Fig 1 shows the regression from the 5 Wikipedia access logs and the previous week ILI to historical ILI data. The regression coefficients were *b*_0_ = 0.0063, *b*_1_ = 17517.3, *b*_2_ = 3206.1, *b*_3_ = 41258.9, *b*_4_ = −71428.7, *b*_5_ = −17410.9, *b*_6_ = 0.955. It is important to note here that the access logs and historical ILI observations used as regressors do not exist on the same scale and therefore it is not correct to use these coefficients to infer importance of the various terms as predictors of ILI. This issue is further complicated by the fact that the individual Wikipedia access logs are not independent or uncorrelated with each other.

### Model description

We only model the U.S. ILI data during the part of the year that we designate as the *influenza season*. Our forecasts are ordered by *epidemiological week* (also called CDC week or MMWR week) since this is how ILI data are reported by the CDC [48]. Epidemiological weeks are used throughout public health reporting in the U.S. (and many other regions of the world). Their widespread acceptance makes them a natural temporal scale to use in disease forecasting.

**Fig 2:**
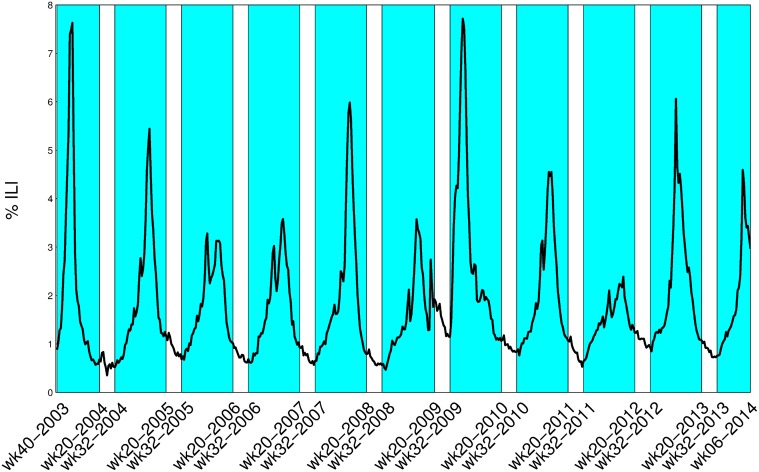
Defining a maximal influenza season. We highlight the weeks corresponding to our maximal influenza season over which we parameterize our forecast. Since our model does not include re-infection or loss of immunity we can only hope to forecast one pre-defined season at a time.

The mathematical model we use for influenza spread does not include re-infection of individuals or loss of immunity and therefore can only hope to model one season’s influenza course. For this reason, it was necessary to define a fixed maximum possible length influenza season that would include the earliest possible ramp up of the flu season and the latest possible tapering off. By examining the ILI data for the entire U.S. from the 2002–2003 season to the 2012–2013 season, it was found that influenza incidence does not start to noticeably increase until at least epidemiological week 32 (mid-August). Moreover, once the influenza peak has passed, the incidence decreases to non-epidemic levels by at least epidemiological week 20 (mid-May). To illustrate our maximal flu season’s relation to historical ILI data we have included Fig 2 with our flu season highlighted. The exception to our maximal influenza season range is the the 2009 H1N1 pandemic, which emerged in the late 2008–2009 season causing this season to be prolonged and an early start in 2009–2010 season. With the flu season defined to be between epidemiological week 32 and epidemiological week 20, even the 2009 H1N1 emergence is mostly accounted for. We emphasize that our definition of a fixed maximal influenza season allows us to avoid modeling influenza prevalence during the dormant summer months, a task which would require some re-introduction of susceptible individuals into the population. It is possible that, through modeling the increase of the susceptible population over the summer months or the change in distinct influenza strains the definition of an influenza season could be made unnecessary.

The U.S. ILI data between mid-August and mid-May are then modeled using a Susceptible-Exposed-Infected-Recovered (SEIR) differential equation model [49–51]. The standard SEIR model is then modified to allow seasonal variation in the transmission rate [52] and to account for heterogeneity in the contact structure [53–55]. We will refer to the model as the *seasonal *S*^*ν*^*EIR** model. This model does not account for several factors that could possibly be important for forecasting influenza dynamics such as spatial disease spread, behavior change due to disease, multiple viral strains, vaccination rates, or more detailed contact structure [56–59].

In our model, the U.S. population is divided into epidemiological categories for each time *t* > 0 as follows: the proportion *susceptible* to flu *S*(*t*), the proportion *exposed* (and noninfectious, asymptomatic) *E*(*t*), the proportion *infectious and symptomatic* *I*(*t*), and the proportion *recovered and immune* *R*(*t*). Since there is usually a single dominant strain each flu season, we assumed that recovered individuals are then immune to the disease for the remainder of the season. In practice, this assumption is not entirely accurate since an individual that contracted and recovered from one strain can get infected from a different strain in the same season [56]. Indeed, in the 2013–2014 influenza season, an elevated level of ILI was maintained well after the primary peak. Upon investigation of World Health Organization and the National Respiratory and Enteric Virus Surveillance System (WHO/NREVSS) strain subtyping data this elevated level correlated with the emergence of influenza B as a secondary dominant strain. We still believe the presentation of our results using a single strain model to be informative especially since one will always have to weigh the cost of model parameter explosion, which can confound identifiability of the model from data with model complexity. After all, an influenza model with no spatial heterogeneity and *n* strains would consist of 2^*n*−1^(*n*+2) independent equations [60] compared to the three independent equations we must identify in (2).

The seasonal *S* ^*ν*^ *EIR* model is defined by the following system of ordinary differential equations:

$$\begin{array}{ccc} \frac{dS}{dt} & = & -\beta \left(t;\beta_{0},\alpha ,c,w\right)IS^{\nu }\\ \frac{dE}{dt} & = & \beta \left(t;\beta_{0},\alpha ,c,w\right)IS^{\nu }-\theta E\\ \frac{dI}{dt} & = & \theta E-\gamma I\\ \frac{dR}{dt} & = & \gamma I\\ S(0) & = & S_{0}\;E\left(0\right)=E_{0}\;I\left(0\right)=I_{0}\;R\left(0\right)=1-\left(S_{0}+E_{0}+I_{0}\right). \end{array}$$ (2)

**Fig 3:**
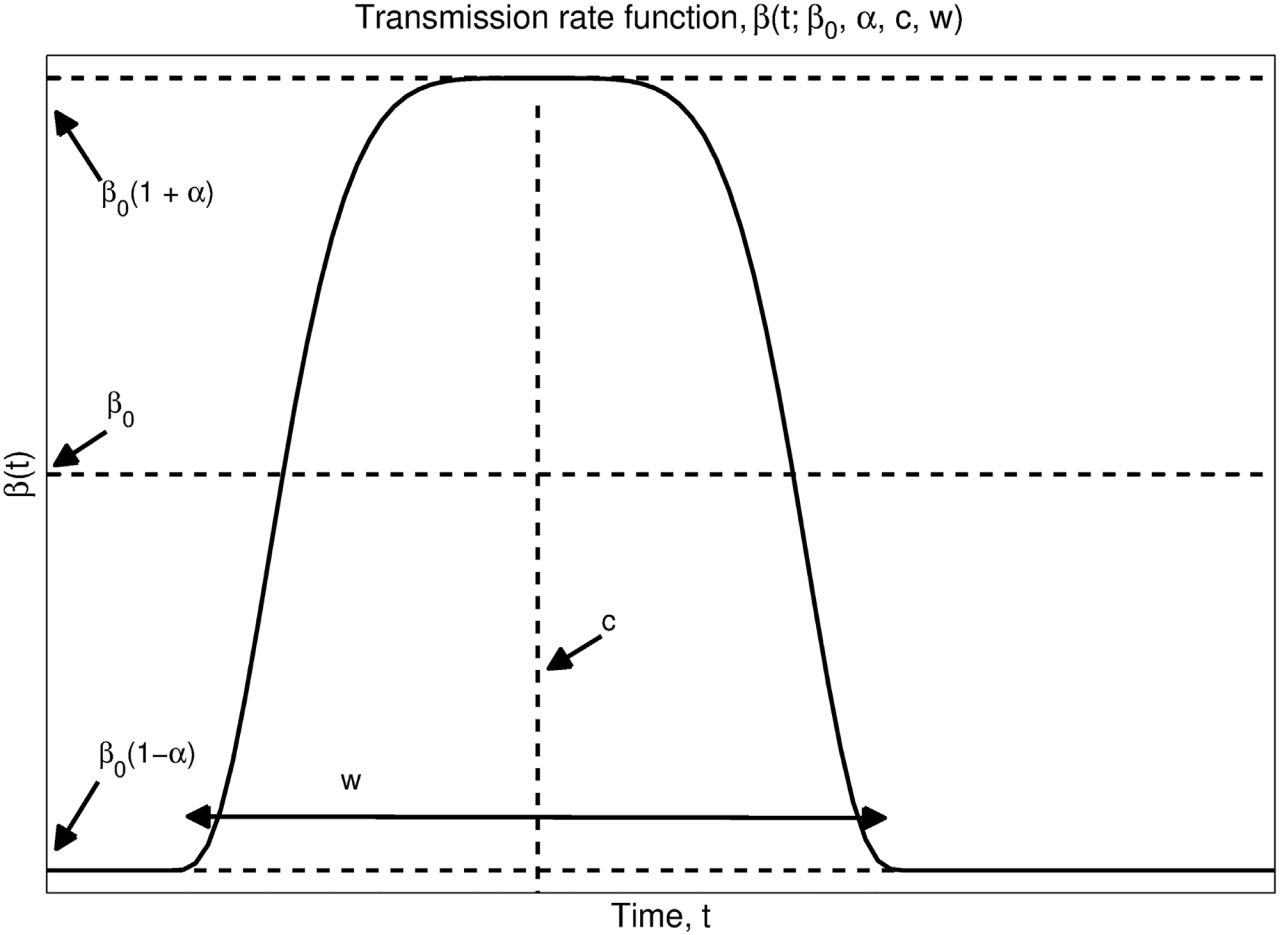
The transmission rate function *β*(*t*;*β*_0_, *α*, *c*, *w*). The transmission function is chosen to be a smooth, five times differentiable, bump function ranging between *β*_0_(1+*α*) at the peak of flu transmission and *β*_0_(1−*α*) at the low point. This is done to account for seasonality in our model. The parameters *c* and *w* control the center (peak of elevated flu transmission) and width (duration of elevated flu transmission).

*S*(*t*), *E*(*t*), *I*(*t*), and *R*(*t*) are the proportions of the U.S. population at time *t* > 0 defined above. Individuals transition from exposed to infectious with constant incubation rate *θ*, and they recover at constant rate *γ*. The transmission coefficient, *β*(*t*;*β*_0_, *α*, *c*, *w*) is allowed to vary over the course of the flu season. The specific variation is controlled by the parameters (*β*_0_, *α*, *c*, *w*), as shown in Fig 3. Algebraically, the transmission rate is defined by *β*(*t*;*β*_0_, *α*, *c*, *w*) = *β*_0_(1+*αf*(*t*;*c*, *w*)) where the smooth bump function, *f*, is defined as

$$\begin{array}{c} f\left(t;c,w\right)=\{\begin{array}{cc} 2{\left(1-{\left|\frac{2(t-c)}{w}\right|}^{5}\right)}^{4}-1, & |\frac{2(t-c)}{w}|<1\\ -1, & \text{otherwise}\;\;. \end{array} \end{array}$$ (3)

The parameters *c* and *w* control the center (peak of elevated flu transmission) and width (length of elevated flu transmission). The max transmission and minimum transmission levels attained are then controlled by *β*_0_ and *α*.

To model some aspects of heterogeneity in the influenza contact network, we use a power-law scaling, *ν*, on the susceptible proportion of the population in the term *S* ^*ν*^. Including this factor has been demonstrated to be an effective approach for this simple model to better fit large-scale detailed agent based models with a heterogeneous contact network [54]. Though, in [54], it was shown that an individual cities’ contact network causes the scaling power *ν* to vary city by city the findings were driven by fitting the power to data. Therefore, we proceed under the hypothesis that there will be a *ν* that provides a best fit to data for the entire U.S. ILI dataset. After optimization of the model to U.S. ILI data this was indeed verified to be the case.

### Prior distribution estimation

We start by specifying a distribution of model parameterizations that we will consider before any observations from the 2013–2014 season are available. This *prior* distribution specifies what we think is possible to observe in the new influenza season. Therefore, it is based on the previously observed ILI data and is broad enough to assign a high likelihood to any of the past influenza seasons. Though our method of specifying a prior is reasonable enough to meet this criterion, it does not rely on more rigorous approaches, such as full Markov chain Monte Carlo exploration of the observation’s influence on an uninformative prior distribution. This will be left to future work.

Let us assume that we have ILI observations from *M* different influenza seasons. The observations for each season are made at regular intervals, Δ*t* = 1week, from the end of epidemiological week 32 to the end of epidemiological week 20 of the following year. With the seasons indexed by *i* we denote these data by

$$\begin{array}{c} d_{1:K}^{i}={\left(d_{\Delta t}^{i},d_{2\Delta t}^{i},\cdots ,d_{K\Delta t}^{i}\right)}^{T}, \end{array}$$ (4)

with *K* being the number of weeks in each season and *i* = 1, 2, …, *M*.

A solution of our model is determined by the parameterization vector

$$\begin{array}{c} p={\left(S_{0},E_{0},I_{0},\beta_{0},\alpha ,c,w,\theta ,\gamma \right)}^{T}, \end{array}$$ (5)

and each choice of **p** yields a discretely sampled solution vector

$$\begin{array}{c} \Psi_{1:K}={\left(\psi_{\Delta t}^{T},\psi_{2\Delta t}^{T},\cdots ,\psi_{K\Delta t}^{T},p^{T}\right)}^{T}. \end{array}$$ (6)

The state of our *S* ^*ν*^ *EIR* model is denoted by

$$\begin{array}{c} \psi_{t}={\left(S(t),E(t),I(t)\right)}^{T}. \end{array}$$ (7)

The link between our epidemiological model and the data is obtained from the infected proportion at the discrete time points. Specifically, it is the model-to-data map defined by

$$\begin{array}{c} M_{p}\left[\Psi_{1:K}\right]={\left(100\cdot I(\Delta t),100\cdot I(2\Delta t),\cdots ,100\cdot I(K\Delta t)\right)}^{T}, \end{array}$$ (8)

with the multiplication changing the proportion into a percentage, which is what ILI is measured in. Our goal now is to determine a prior distribution for **p**, *π*_0_(**p**), so that samples drawn from the prior, make *M*_*p*_[Ψ_1:*K*_] close to at least one prior season’s dataset, $d_{1:K}^{i}$.

For each season’s data, *i* = 1, 2, …, *M*, we can determine an allowable **p** ^*i*^ by approximately solving the non-linear optimization problem

$$\begin{array}{c} p^{i}=\underset{p}{arg min}{∥d_{1:K}^{i}-M_{p}\left[\Psi_{1:K}\right]∥}^{2}, \end{array}$$ (9)

where ‖⋅‖ denotes the root sum of squares discrepancy over the discrete time points. The approximate solution to (9) is reached by applying a stochastic optimization algorithm [61, 62] and this process is repeated *L* times for each season. Variation in these optimal **p** for a single season are considered to represent variation that we should allow in the prior distribution of our model. This process then yields *M*⋅*L* approximate solutions ${\text{p}}_{l}^{i}$, which are then treated as samples from a prior distribution for the model’s parameterization.

A log-normal distribution, fit to these samples, is chosen for *π*_0_(**p**). We have chosen a log-normal distribution for *π*_0_(**p**) since physically all terms in **p** must be positive and the relation of the log-normal to a Gaussian distribution makes it a convenient choice when implementing our ensemble Kalman filtering method.

### Data assimilation

An iterative data assimilation process is implemented to continually adjust the parameters and initial state of the seasonal *S* ^*ν*^ *EIR* model, which incorporates new ILI and Wikipedia observations. The model can then be propagated through the end of flu season to create an informed forecast. Data assimilation has been successful in a diverse array of fields, from meteorology [20, 21] to economics [22] but has only recently begun to be applied to disease spread [28–30, 32, 33, 35–37]. One of the most common schemes for data assimilation is based on the Kalman filter in which both the model error and observation error are assumed to be Gaussian and all mathematical models are assumed to be linear. Updating the model using observations is then accomplished by conditioning a joint Gaussian distribution. In particular, the ensemble Kalman filtering methods we use here, explained in detail below, have been successfully applied to non-linear systems of ordinary differential equations with a much higher degree of non-linearity and a much higher dimension than our *S* ^*ν*^ *EIR* model [63–65]. Our approach to using Kalman filtering to estimate the underlying parameters of our model is more difficult than an estimation of the state of the system. However, parameter estimation too has had success with much larger and more non-linear systems of differential equations [66].

We use an ensemble Kalman smoother (enKS) [21, 67], with propagation always performed from the start of the influenza season, to assimilate the ILI/Wikipedia data into the transmission model. Our implementation of the enKS directly adjusts only the parameterization of our system. However, the adjustment is determined using information about the model’s dynamics throughout the season.

With the exception of reference [45], the previous disease forecasting methods only use the most recent observation to update the epidemic model. This can lead to problems in determination of the underlying model parameters since the dynamic trends of the data are not considered during the model adjustment. The enKS method we use is more sensitive to the underlying dynamics of the data timeseries.

When performing data assimilation to adjust the current model state, conditions such as the population in each epidemic category summing to the total population are often disrupted. Moreover, if the model state is adjusted directly each time an observation is made, the forecast epidemic curve may not represent any single realization of the epidemic model. This makes it difficult to judge systematic model error and thus identify specific areas where the model may be improved. In our assimilation scheme only the model’s parameterization and initialization are adjusted. Therefore, each forecast represents a realization of the model.

For a more precise description of the ensemble Kalman smoother implemented for this work we refer the reader to [21]. The main idea is to view the time series of our epidemiological model together with its parameters and the ILI/Wikipedia data as a large Gaussian random vector. We can then use standard formulas to condition our *S* ^*ν*^ *EIR* time series and parameters on the ILI/Wikipedia observations. Draws from this conditional Gaussian give an updated parameterization of the system from which we can re-propagate to form an updated forecast.

The enKS is similar to a standard ensemble Kalman filter except that instead of just using the most recent data to inform the forecast it uses a number of the most recent observations. The three most current observations, including Wikipedia observations, are used to inform our forecasts. The advantages of using the enKS is that more of the current trends/dynamics of the observations are used in each assimilation step. This helps in estimating the underlying parameterizations of the system by propagating the observation’s information backward into the model ensemble’s history [21, 67].

For each week in the simulation, we receive the ILI data and a Wikipedia estimate of the ILI data the following week. These data become available at regular time intervals of Δ*t* = 1week and we denote the data corresponding to the first *K* weeks by *d*_1:*K*_ as in (4). Note that now the index *K* corresponds to the most current week instead of the last week in the season. During the data assimilation step, *d*_1:*K*_ is compared with simulations of our *S* ^*ν*^ *EIR* model and its parameterization, sampled at weekly intervals, denoted by Ψ_1:*K*_ as in (6) above.

The link between our epidemiological model and the data is again obtained from the simulated infected proportion at the time the most recent data are collected, *K*Δ*t*. This is similar to (8) except that we only use the infected proportions corresponding to recent data. Specifically, the *model-to-data map* is

$$\begin{array}{c} M\left[\Psi_{1:K}\right]={\left(100\cdot I((K-2)\Delta t),100\cdot I((K-1)\Delta t),100\cdot I(K\Delta t)\right)}^{T}. \end{array}$$ (10)

Using this model-to-data map implies that we are only attempting to model and forecast the dynamics of ILI as opposed to the actual proportion of the U.S. population infected with influenza. The last three sampled values of the infected proportion are used, corresponding to the Wikipedia estimated ILI, the most current ILI, and the previous week’s ILI observations.

In the ensemble Kalman filtering framework, the simulation and data, ${\left(\Psi_{1:K}^{T},d_{(K-2):K}^{T}\right)}^{T}$, are assumed to be jointly Gaussian distributed. Therefore, the conditional random vector Ψ_1:*K*_∣*d*_(*K*−2):*K*_ is also Gaussian, which we can sample from. We only sample the marginal distribution, which is also Gaussian, of our *S* ^*ν*^ *EIR* parameterization, **p**∣*d*_(*K*−2):*K*_. Samples of **p**∣*d*_(*K*−2):*K*_ are then used to re-propagate our *S* ^*ν*^ *EIR* model from an adjusted initial state to form an updated forecast. When new data are collected on the (*K*+1)^th^ week, the process is repeated.

The remaining details of the enKS implementation deal with the choice of the mean and covariance structure of the joint Gaussian distribution for ${\left(\Psi_{1:K}^{T},d_{(K-2):K}^{T}\right)}^{T}$. Our implementation followed Evensen’s explanation [21]. In short, the mean is determined by sampling our *S* ^*ν*^ *EIR* model at different parameterizations, while the covariance structure is determined by assumptions on the observational error for ILI, the Wikipedia estimate, and our epidemiological model.

### Evaluating forecast accuracy

To evaluate the accuracy of a forecast, we compare the distribution determined by the ensemble with the actual observed disease data. We can, of course, only perform this evaluation retrospectively since we require data to evaluate our forecasts against. Since the enKS method assumes that the forecast distributions are Gaussian, we can evaluate the forecast’s precision by scaling the distance of our forecast mean from the observation using the ensemble covariance. Such a distance has been widely used in statistics and is commonly referred to as the *Mahalanobis distance* (M-distance) [68]. The M-distance gives a description of the quality of the forecast that accounts for both precision in the mean prediction and precision in the dispersion about the mean. Other methods of evaluating forecast accuracy such as the root mean square error only consider how close the mean of the forecast is to the observations. Thus, a distribution with a great deal of uncertainty, or dispersion, can have a small root mean square error compared to observations.

A forecast is made up of an analysis ensemble of parameterizations ${\left\{{\text{p}}_{K}^{i}\right\}}_{i=1}^{N}$, *K* is the index corresponding to the most recently assimilated observation and *N* is the size of the ensemble. Each ${\text{p}}_{K}^{i}$ is drawn from a Gaussian distribution conditioned on the most recent observations as described above. We can form a forecast of ILI data for the entire season by propagating the ${\text{p}}_{K}^{i}$ through our *S* ^*ν*^ *EIR* model. We will denote the discretely sampled time series of these realizations by $\psi_{K}^{i}$. The M-distance will then be evaluated using the ensemble of forecast observations, ${\left\{M_{f}[\psi_{K}^{i}]\right\}}_{i=1}^{N}$, corresponding to the infected proportion time series, after the time index *K*, with each $\psi_{K}^{i}$ scaled to a percentage.

The M-distance is then calculated from the $M_{f}[\psi_{K}^{i}]$ using their sample mean and covariance denoted *μ*_obs_ and *C*_obs_, respectively. Letting ${\tilde{d}}_{K}$ correspond to un-assimilated observations (i.e., observations with time indices greater than *K*), the M-distance we evaluate is

$$\begin{array}{c} \rho \left({\tilde{d}}_{K},\left\{M_{f}\left[\psi_{K}^{i}\right]\right\}\right)=\sqrt{{\left({\tilde{d}}_{K}-\mu_{\mathrm{obs}}\right)}^{T}C_{\mathrm{obs}}^{-1}\left({\tilde{d}}_{K}-\mu_{\mathrm{obs}}\right)}. \end{array}$$ (11)

In order to judge the quality of our forecasting methods and ultimately to justify the complexity of our data assimilation procedure, we generate a simplistic *straw man* model for comparison. We first collect all historical time series of disease outbreaks and then determine a correspondence time between each of the time points for each of the outbreak datasets. This gives a common time frame for each of the historical data sets. Then, at each of these common time points, the average and standard deviation of the historical observations can be computed. Thus, the straw man forecast consists of a normal distribution at each corresponding time point in the forecast with an averaged mean and standard deviation. We can then evaluate the straw man’s accuracy using the metric given in Eq (11). Given the simple construction of the straw man forecast, this provides a good baseline to necessarily beat, in terms of smaller M-distance, for any compartmental data assimilation-derived forecast. To illustrate the improvement in the M-distance metric of our data assimilative forecast over the straw man forecast, we will calculate the percent of growth (or decline) of the M-distance for the data assimilative forecast in the straw man forecast for each week of the forecast.

Besides using a strictly quantitative measure of forecast accuracy, we also suggest computing more qualitative measures of accuracy. With the ensemble forecast, the samples $\psi_{K}^{i}$ can be used to estimate quantiles of the forecast distribution such as the standard *5-number summary* of the distribution given by the 5%, 25%, 50%, 75%, and 95% quantiles for the seasonal *S* ^*ν*^ *EIR* realizations. Moreover, if we are interested in the forecast of some other quantity of interest derived from the time series of observations, such as the epidemic’s peak time, peak level, duration, or start time, we may also derive 5-number summaries for these quantities by computing the appropriate quantity of interest. Analyzing where the actual observations fall compared to the 5-number summary provides a qualitative way to understand the accuracy of the forecast.

For our data assimilative forecast, we calculate weekly 5-number summaries for four quantities of interest: the start of elevated ILI data, the duration that the ILI data will remain elevated, the timing of the peak of ILI data, and the height of the ILI peak. Here, the start of the influenza season is defined to be the first week that ILI goes above 2% and remains elevated for at least 3 consecutive weeks. The end of the influenza season, used to calculate the duration, is when ILI goes below the 2% national baseline and remains there.

## Results

We present the results of our prior estimation techniques and our evaluation of forecast accuracy for the 2013–2014 influenza season. Again, to restrict our forecast to only one seasonal outbreak and avoid modeling influenza dynamics during the dormant summer months, our maximal influenza season is defined to be between the 32^nd^ and 20^th^ epidemiological weeks, or from mid-August to mid-May, for successive years. Our forecasts are only valid during this time period.

### Prior forecast

**Fig 4:**
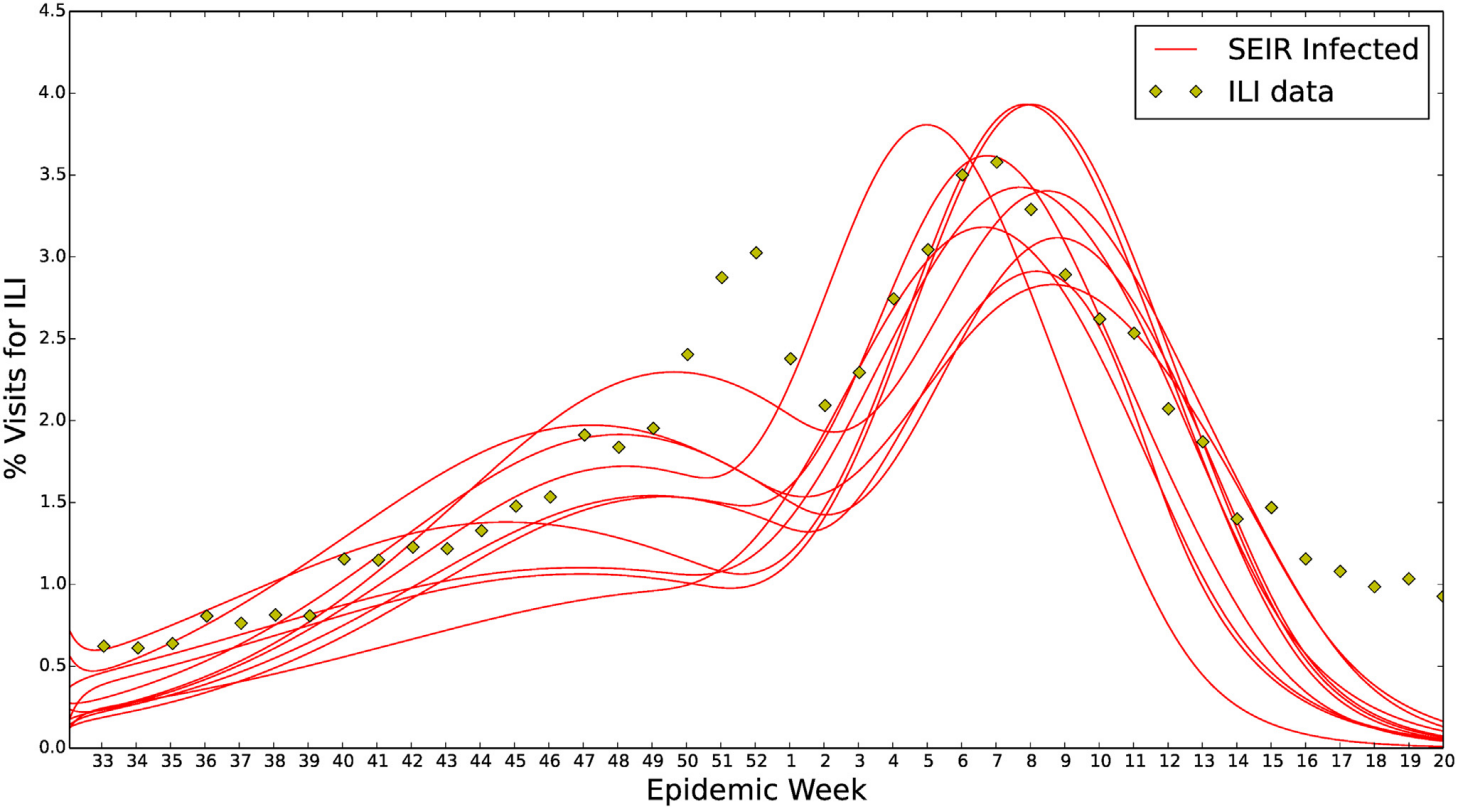
Seasonal *S* ^*ν*^ *EIR* fit to 2006–2007 U.S. ILI data. Ten seasonal heterogeneous *S* ^*ν*^ *EIR* model parameterizations for the U.S. ILI 2006–2007 data. These are approximate solutions to (9). For each of the influenza seasons, from 2003–2004 through 2012–2013, fits similar to the above were generated. These parameterizations formed the basis for our prior, *π*_0_(**p**). This is a good example of the seasonal *S* ^*ν*^ *EIR* model’s two areas of systematic divergence. In the weeks 50–1 there is a first peak that the model does not catch. However, the fitted model does envelop the secondary peak around the 8^th^ epidemiological week. During the tail weeks 15–20 our *S* ^*ν*^ *EIR* model tapers too quickly.

Historical ILI data from the 2003–2004 U.S. influenza season through the 2012–2013 influenza season were used to generate our prior distribution of the seasonal *S* ^*ν*^ *EIR* model’s parameterization. This was done following the methods described above. An example fit using a stochastic optimization algorithm to find 10 approximate solutions to (9) for the 2006–2007 ILI data is shown in Fig 4. Two things can be noticed from this fit of our epidemiological model. First, there is often a small early peak in the ILI data before the primary peak and our model does a poor job of capturing this. In several conversations we’ve had with experts, the hypothesis has been put forward that the double peak in U.S. ILI data is due to under reporting during the holiday season in the U.S. However, from our observations the first peak can vary in its timing substantially and does not seem to be always present or strongly correlated with the holidays or emergence of separate influenza strains. Second, the ILI data usually remain elevated longer than our model’s realizations can support. Under retrospective examination of the tail for the 2013–2014 season, the extended elevated ILI level appears to be due to the emergence of a secondary dominant strain. Further examination of this phenomenon using historical ILI data is necessary but beyond the scope of this study. Both of these areas point to systematic divergence of the model from data. It is always possible that our model fit is representative of influenza prevalence but, due to bias in the ILI reporting, diverges from the historical data. However, without a stronger ground truth dataset to support this, such hypotheses are difficult to test.

**Fig 5:**
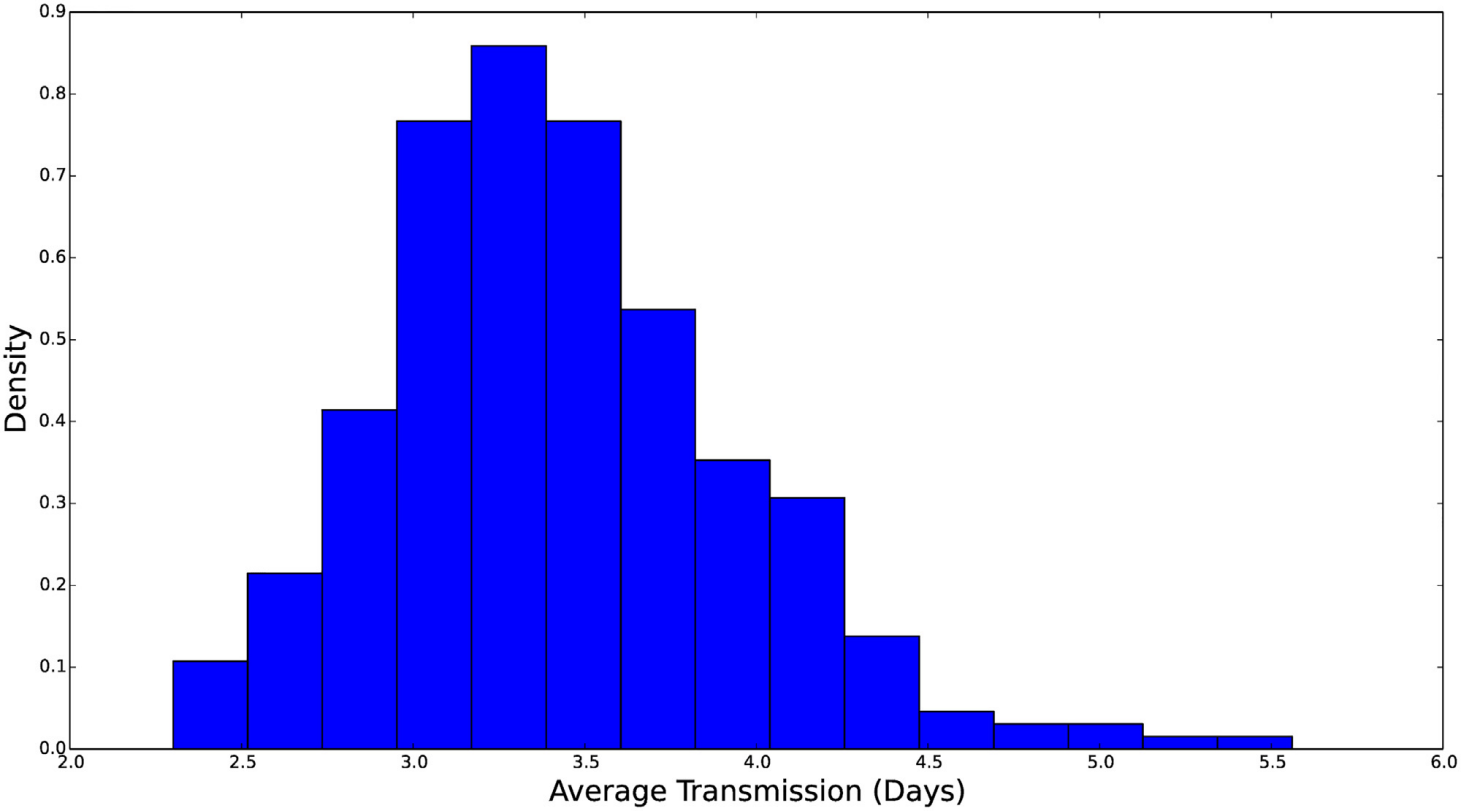
Histogram of the marginal distribution for the average transmission time, measured in days. The rate parameter, *β*_0_, is then the inverse of this average time. We see that this distribution is concentrated over 2–4.5 days. All histograms were generated from 300 samples of *π*_0_(**p**).

**Fig 6:**
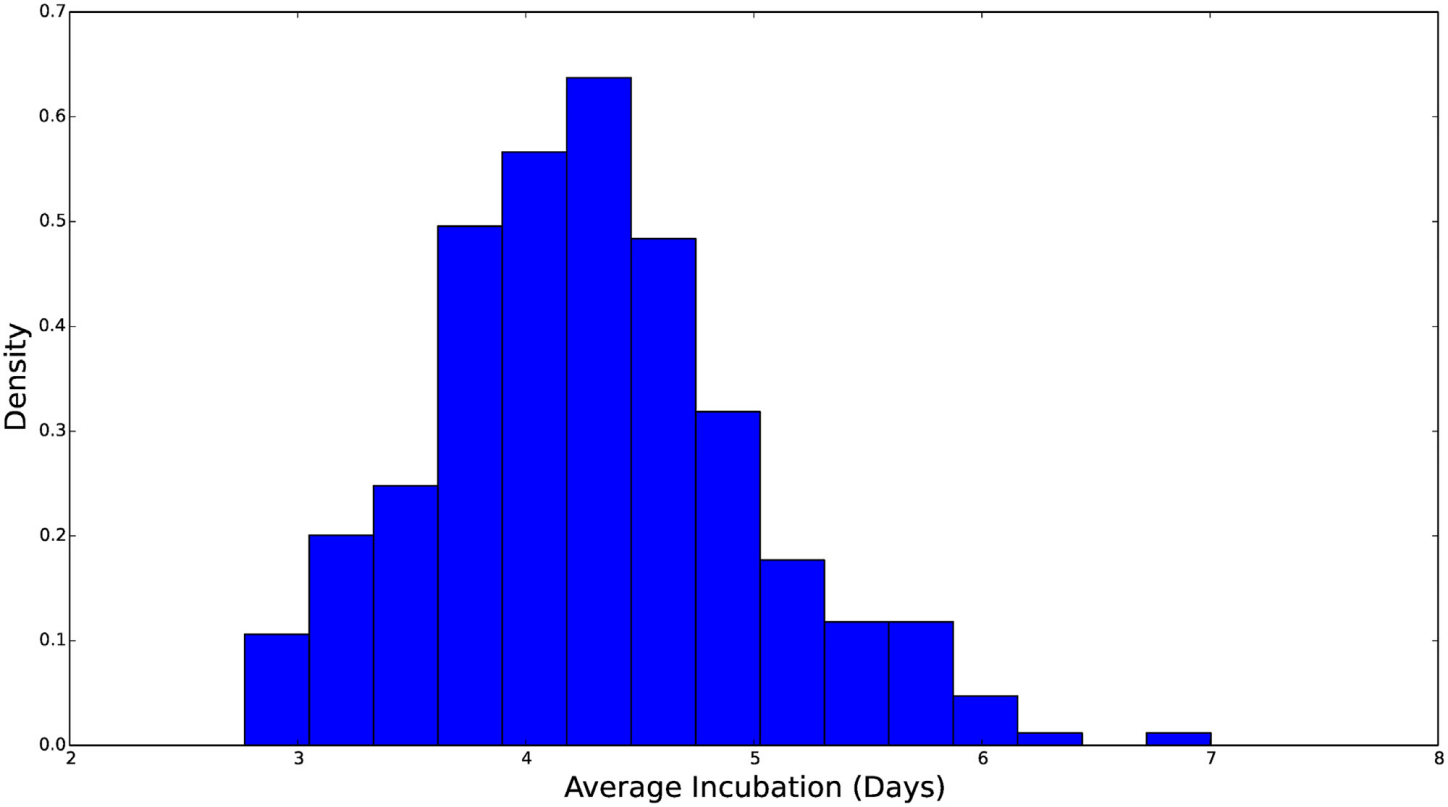
Histogram of the marginal distribution for the average incubation time, measured in days. The rate parameter in our *S* ^*ν*^ *EIR* model, *θ*, is then the inverse of this average time. We see that this distribution is concentrated over 3–6 days and skewed toward longer incubation times.

**Fig 7:**
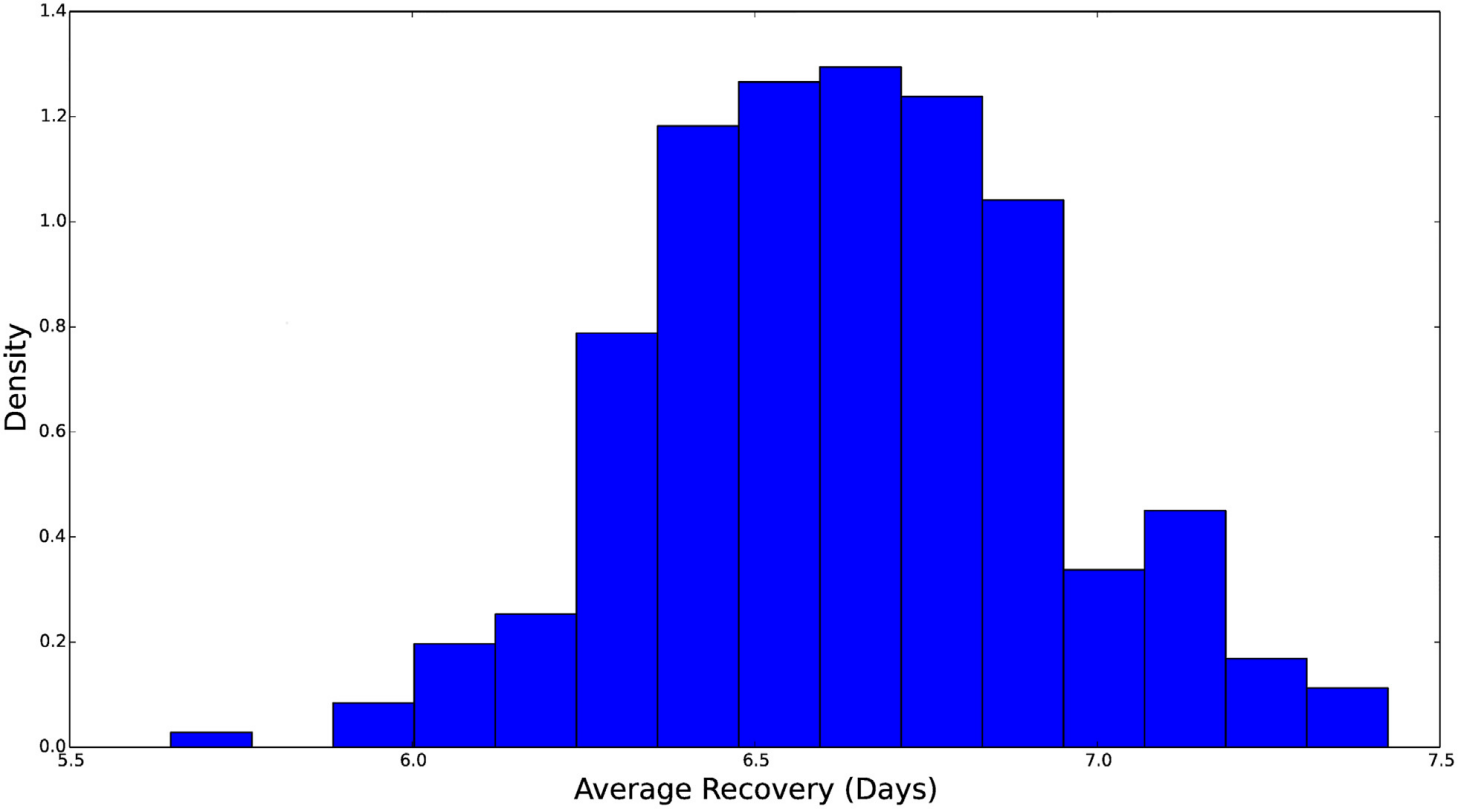
Histogram of the marginal distribution for the average recovery time, measured in days. The rate parameter in our *S* ^*ν*^ *EIR* model, *γ*, is the inverse of this average time. We see that this distribution is concentrated over 6–7 days and skewed toward longer incubation times. The prior distribution for *γ* is more concentrated than the distributions for *θ* and *β*_0_ which means that the ILI data determine this parameter more exactly.

**Fig 8:**
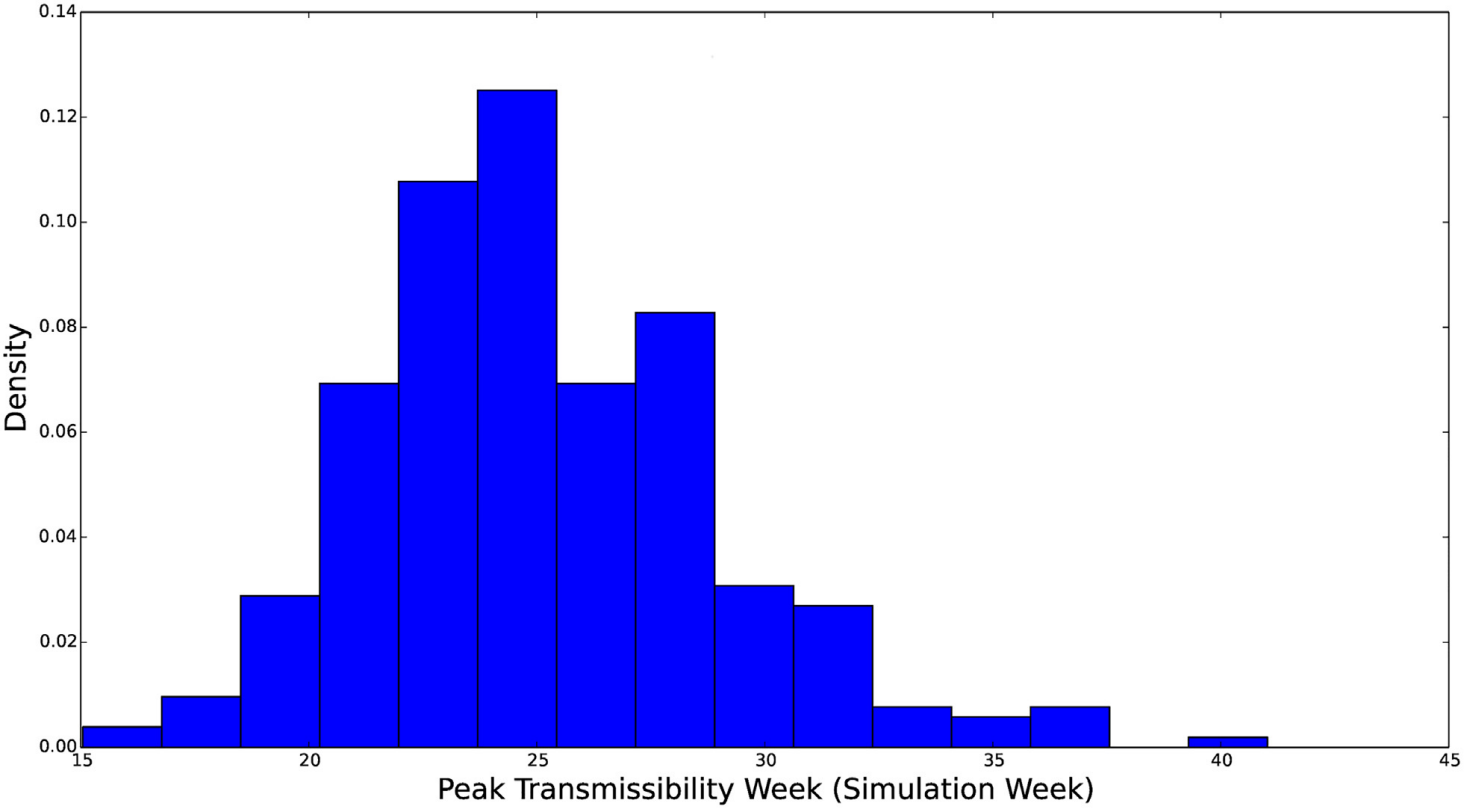
Histogram of the marginal distribution for the peak of the transmissibility function, *β*(*t*;*β*_0_, *α*, *c*, *w*). The parameter *c* here is represented in *weeks since the beginning of simulation*. Thus, a value *c* = 16 corresponds to the peak transmissibility during the 48^th^ epidemiological week. We see that this distribution is concentrated over 20–30 weeks into the simulation and skewed toward late in the simulation.

**Fig 9:**
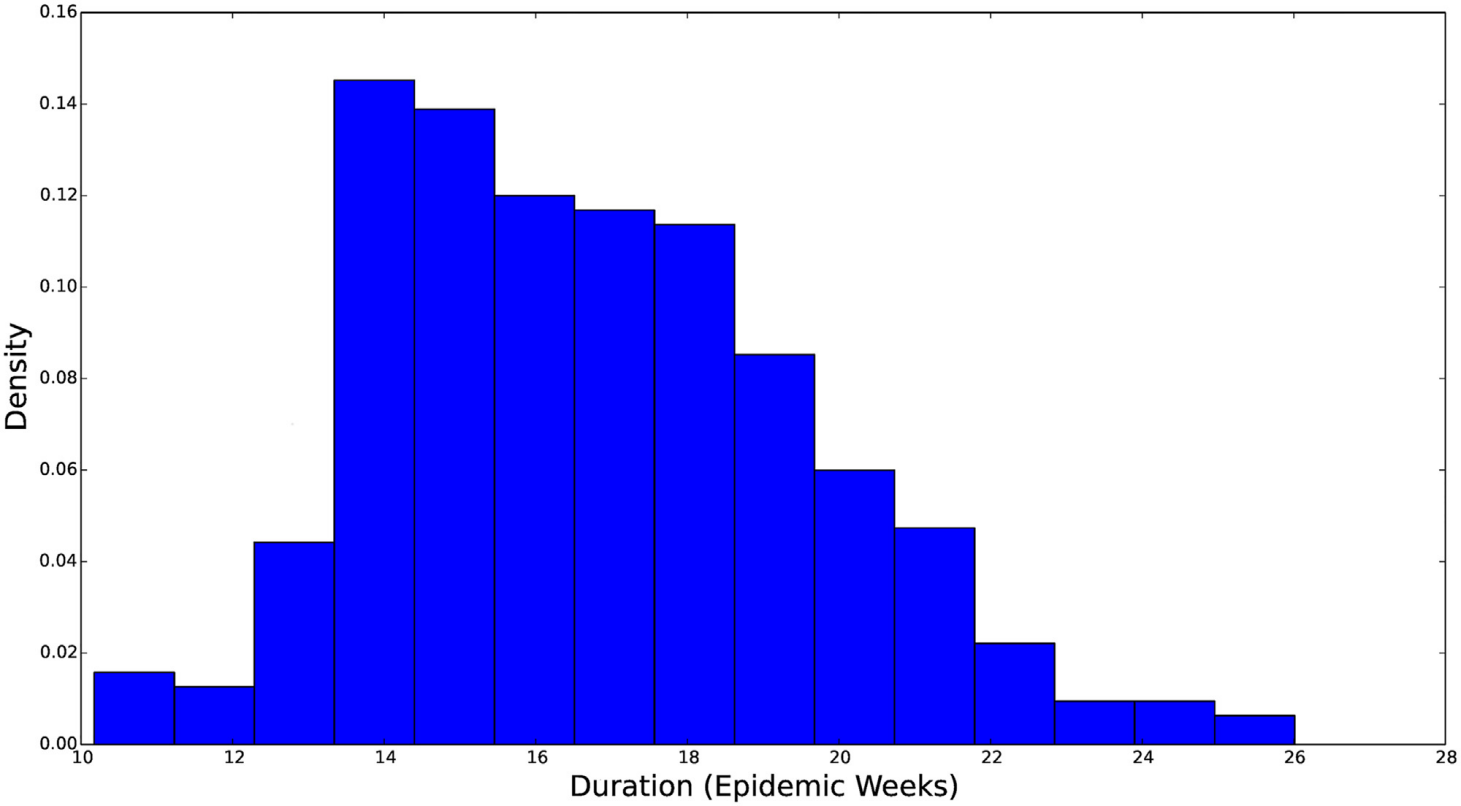
Histogram of the marginal distribution for the duration of heightened transmissibility. The parameter *w* is represented in weeks. A value *w* = 14 corresponds to 16 weeks of elevated transmission. We see that this distribution is concentrated over 14–20 weeks and skewed toward longer periods of elevated transmission.

From the joint prior, *π*_0_(**p**), we can examine samples from the marginal priors to examine our method’s forecast for traits of the *average* influenza season. In Figs 5, 6, 7, 8 and 9 we show histograms of samples from a few of these marginal priors. We see that our methods have determined that the average base time of transmission is 2–5 days, the average incubation time is 3–7 days, and the average recovery time is 6–8 days. This automatically lets us know that the recovery rate is tightly specified by our prior whereas the base transmission and incubation rates are not. Since our *S* ^*ν*^ *EIR* model includes a variable transmission rate, we also include the marginals for the week of peak transmissibility, *c*, and the duration the transmission rate is elevated, *w*. For our prior, the duration *w* is centered over 14–20 weeks into our simulation, while the center *c* is between 20–30 weeks into our simulation. This corresponds to the center of our elevated transmission being between epidemiological week 52 of 2013 and epidemiological week 10 of 2014, between the last week of December and the first week of March.

**Fig 10:**
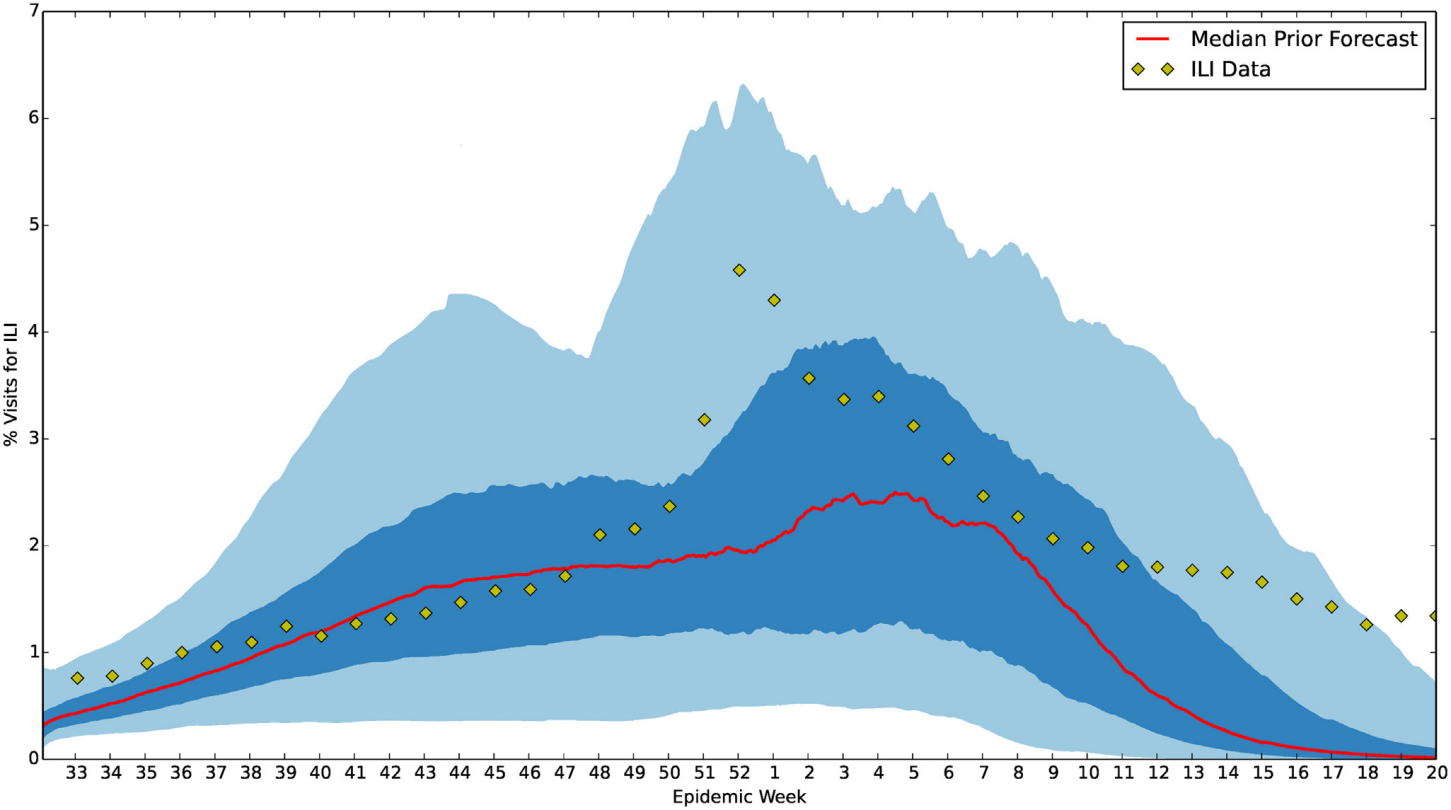
U.S. ILI prior forecast for 2013–2014 flu season. This figure shows the prior forecast along with the 2013–2014 ILI data. Note the potential for an early and late peaking influenza season. The red line represents the median forecast from 300 samples of the prior. The dark blue and light blue regions represent the 50% and 90% credible regions centered around this median, respectively. Credible intervals were also generated from 300 samples of the prior.

Sampling 300 parameterizations from the log-normal prior *π*_0_(**p**) leads to the prior forecast for the 2013–2014 influenza season shown in Fig 10. One can notice that our prior forecast allows for a wide range of peak times and sizes. In general, the earlier the peak, the smaller its forecast height. It is also apparent that our forecast tapers off quickly after the peak occurs. Unfortunately, in carrying out this process of model fitting and estimation of a log-normal prior, it becomes apparent that there is a strong negative bias in our prior forecasts. Our hypothesis for this outcome is that in a typical influenza season, there are many more low ILI observations than there are high ILI observations. Since, in our fitting of the *S* ^*ν*^ *EIR* model, all the ILI data was given equal weight a good fit can be obtained from a model solution that stays below the peak. The effect of this negative bias can be seen in our data assimilative forecasts as a strong negative bias.

### Forecast analysis

**Fig 11:**
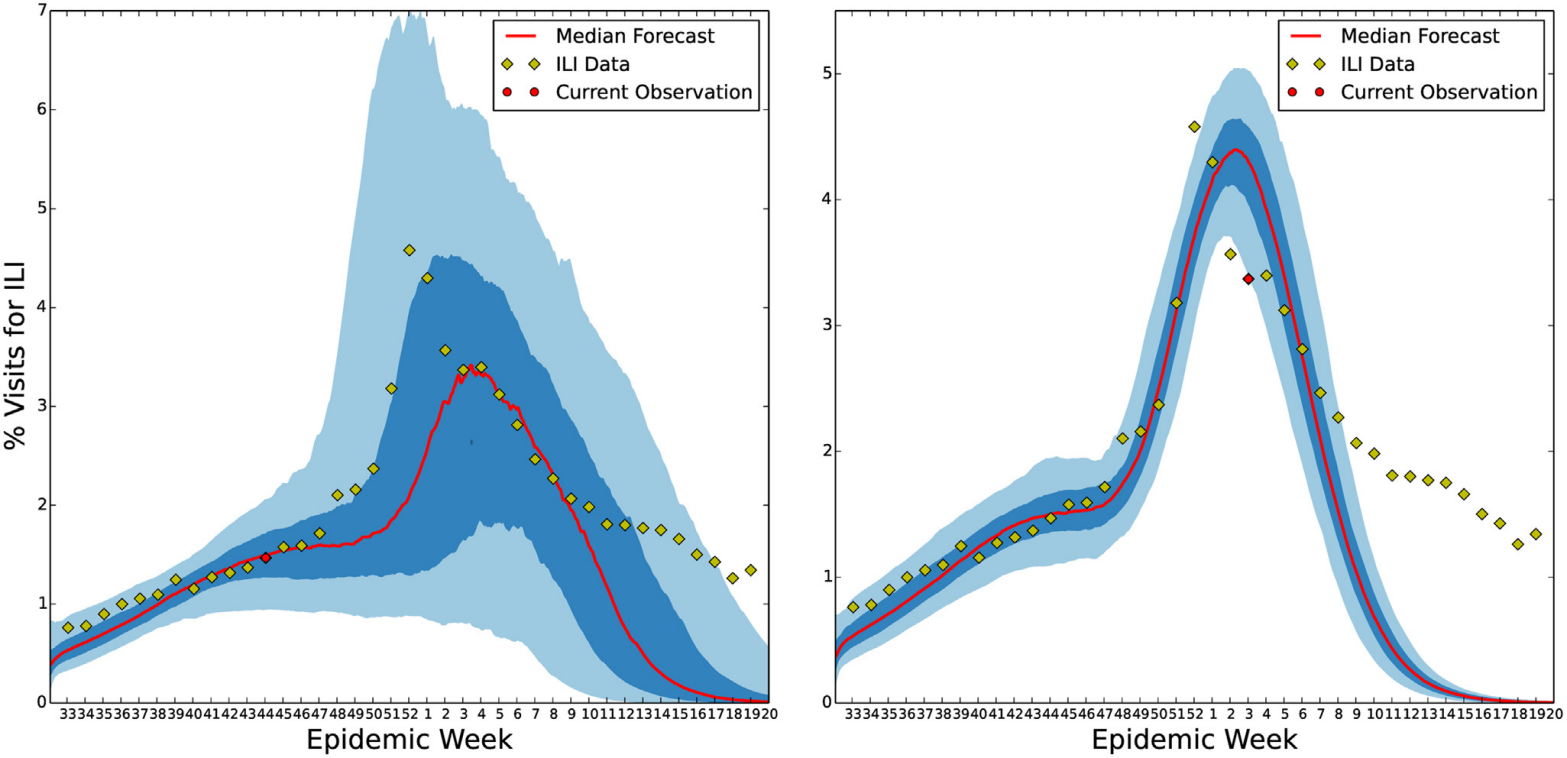
U.S. ILI forecast for the 2013–2014 flu season made during the 43^rd^ (left) and 2^nd^ (right) epidemiological weeks. In each plot the dark blue region represents the region centered about the median in which 50% of forecasts fall, the light blue region represents where 90% of forecasts fall, and the red line represents the median forecast. The diamonds represent the 2013–2014 ILI data with the current data point marked by a red circle.

**Fig 12:**
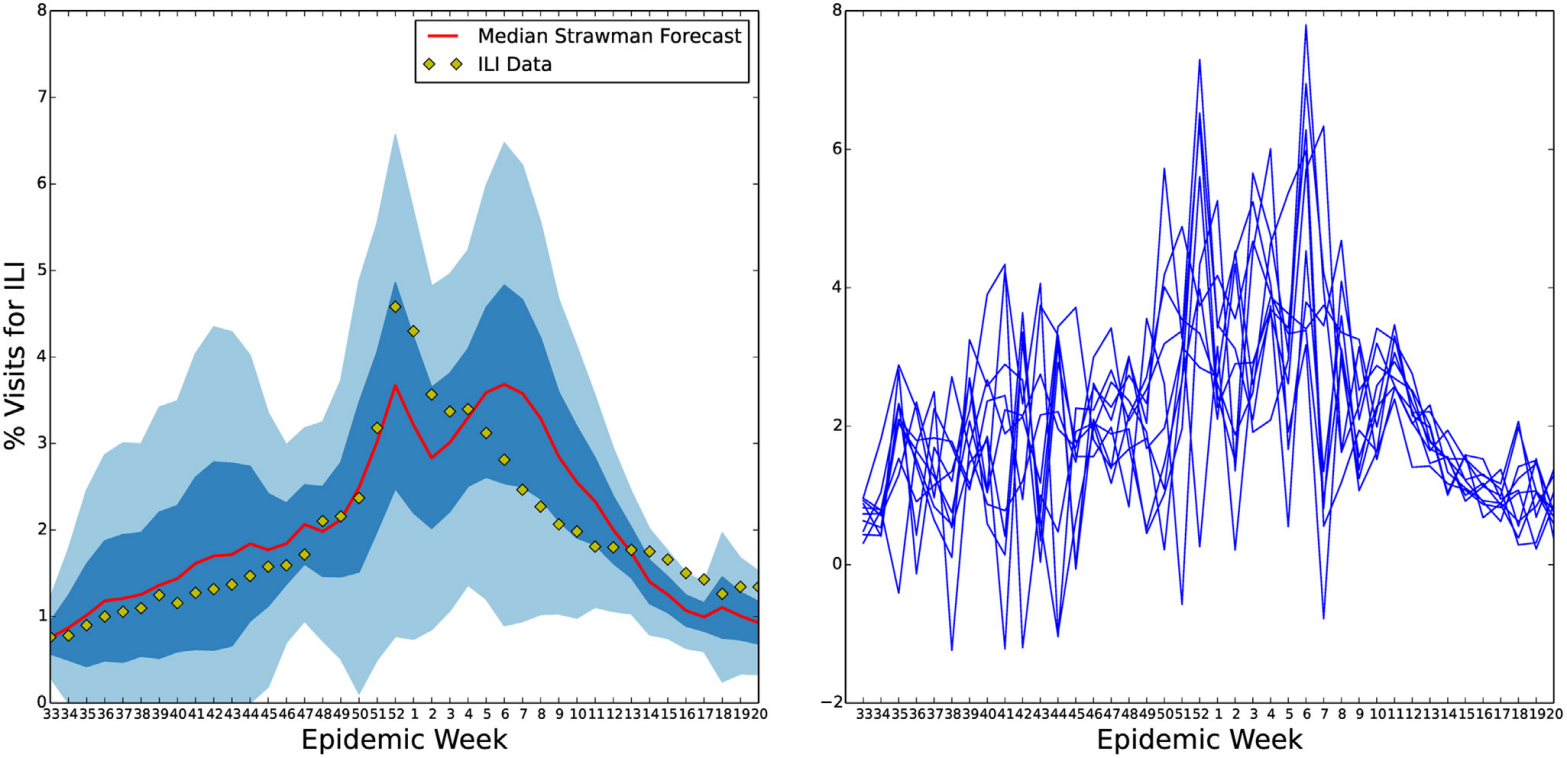
U.S. ILI straw man forecast. This figure shows the results of our straw man prediction based on averaging past flu seasons. Since this forecast of the 2013–2014 influenza season was made using only the statistics of the sample mean and sample standard deviation from previous season’s ILI observations it’s credible intervals (left) do a good job of containing the current influenza season. This forecast does not include any causal model of influenza spread. There is, therefore, no correlation between the forecast at successive time points. This is seen when sampling time series from this forecast (right). The lack of correlation in forecasts for successive weeks severely limits the usefulness of such a forecast for public health decision making. For instance, after the 2013–2014 ILI peak was observed a further ILI peak is forecast to be just as likely six weeks later.

In Fig 11, we show the results of our forecasting process for two different weeks during the 2013–2014 influenza season. Note, the performance of the seasonal *S* ^*ν*^ *EIR* model is drastically reduced once the peak of the influenza season has passed. However, before the peak, the model forecasts a range of possible influenza scenarios that include the 2013–2014 season. In Fig 12, we show the forecast resulting from the straw man approach for the 2013–2014 influenza season. In this section, we analyze both of these forecasts’ performance using the measures described above.

Forecast results illustrated in Fig 11 may seem disappointing at first glance. The variance in the forecast at epidemiological week 43 is quite large and the mean forecast underestimates the peak. For the forecast made on epidemiological week 2, even the 90% credible region of the forecast diverges from the tail of the epidemic despite the addition of many more ILI observations. As discussed above, the underestimation of the forecast is most likely due to a bias toward low ILI seasons in the prior forecast we have used. In this way, the use of the enKS actually is a success since by our week 43 forecast the projected ILI peak is much higher than the median peak predicted in our prior distribution. In other words, the data assimilation method is performing well, but the prior and possibly the model itself has significant bias that needs to be corrected in future work. Model bias (i.e., systematic divergence of the model from ILI observations) is also responsible for the degradation of performance in our forecast once the ILI peak has past. Our *S* ^*ν*^ *EIR* model has a difficult time simulating an elevated tail. Thus, there are fewer model parameterizations that are close to ILI observations by the 2^nd^ epidemiological week, so the variance in our forecast ensemble is reduced even though accuracy is decreased.

Judging accuracy for forecasts is difficult to do from figure such as those in Figs 11 and 12. For instance, in Fig 12, the 2013–2014 season is mostly included in the 50% credible interval. However, individual forecasts made from the straw man method look different, as time series, from an ILI season since little of the dynamics of ILI are present in the straw man model. On the other hand some of the 2013–2014 ILI data falls outside the 50% credible region during the 43^rd^ epidemiological week forecast in Fig 11. As discussed previously, this is most likely due to error in the original model and the bias toward low influenza seasons in our prior. However, the accuracy of a probabilistic forecast like Fig 11 should always be in terms of probability. It is possible that the 2013–2014 ILI data given the 2013–2014 ILI data up to epidemiological week 43 should have been assigned a lower probability based on previous seasons. In particular, based on previous ILI seasons it is not uncommon to see a much lower and later ILI peak given the ILI level at epidemiological week 43 for 2013–2014. For this reason, probabilistic forecasts are difficult for public health planners to rely on. However, without a forecast that predicts the dynamics of ILI faithfully week by week, planning can not be informed from the forecast.

#### Quantitative accuracy

**Fig 13:**
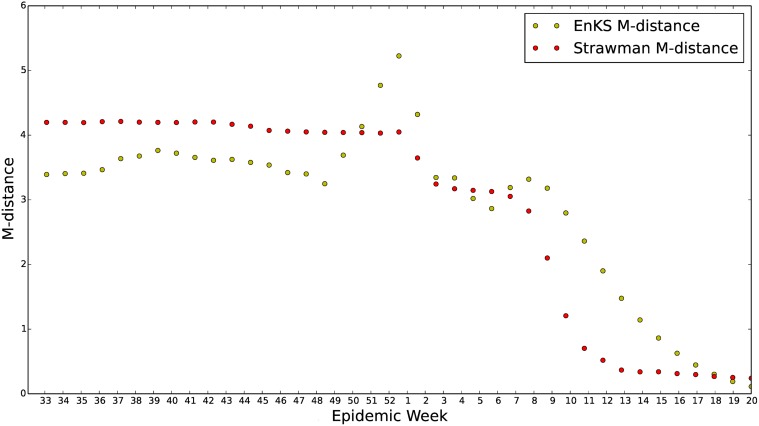
*S* ^*ν*^ *EIR* with enKS vs. straw man forecast for the 2013–2014 U.S. ILI data. The M-distance between U.S. 2013–2014 ILI data and the two forecasts is plotted. The M-distance between the forecast and ILI data is calculated for each epidemiological week until the end of the influenza season. The M-distances at week 36 uses the forecast observations from week 36 of 2013 to week 20 of 2014 and the ILI data from week 36 of 2013 to week 20 of 2014. The M-distances plotted for the straw man prediction use sample covariances and means calculated from 300 time series draws of the straw man forecast. Due to the lack of causal relations included in the straw man model this measure of accuracy is significantly lower in the early season for the straw man prediction. This figure shows that the data assimilation forecast has a noticeably smaller M-distance, and therefore is quantitatively better, than the straw man model for the early influenza season. Once the influenza season peaks the success of the forecast breaks down due to model error. It is interesting to note that due to the enKS data assimilation our *S* ^*ν*^ *EIR* forecast seems to attempt self-correction, i.e. the M-distance is increasing and then decreases.

In Fig 13, we show the successive M-distances computed for our forecast and for 300 samples from the straw man forecast. We notice that until the peak of the 2013–2014 influenza season, the data-assimilative forecasting scheme has a noticeably smaller M-distance than that of the straw man forecast. However, after the peak of the influenza season, during week 52 for 2013–2014, the straw man shows considerably smaller M-distance. This is due to the seasonal *S* ^*ν*^ *EIR*’s inability to taper off slowly from the peak of the flu season. After the peak, our model has exhausted its susceptible proportion of the population, and the infected proportion rapidly goes to zero. A possible point of confusion in this analysis is the sharp drop off of the M-distance as the end of our forecast horizon is approached. This is due to the decreased dimension of the data being forecasted. During week 17, the forecasts only need to predict the ILI data for 3 more weeks and distances in this 3-dimensional space grow much slower as a function of week-by-week error.

**Fig 14:**
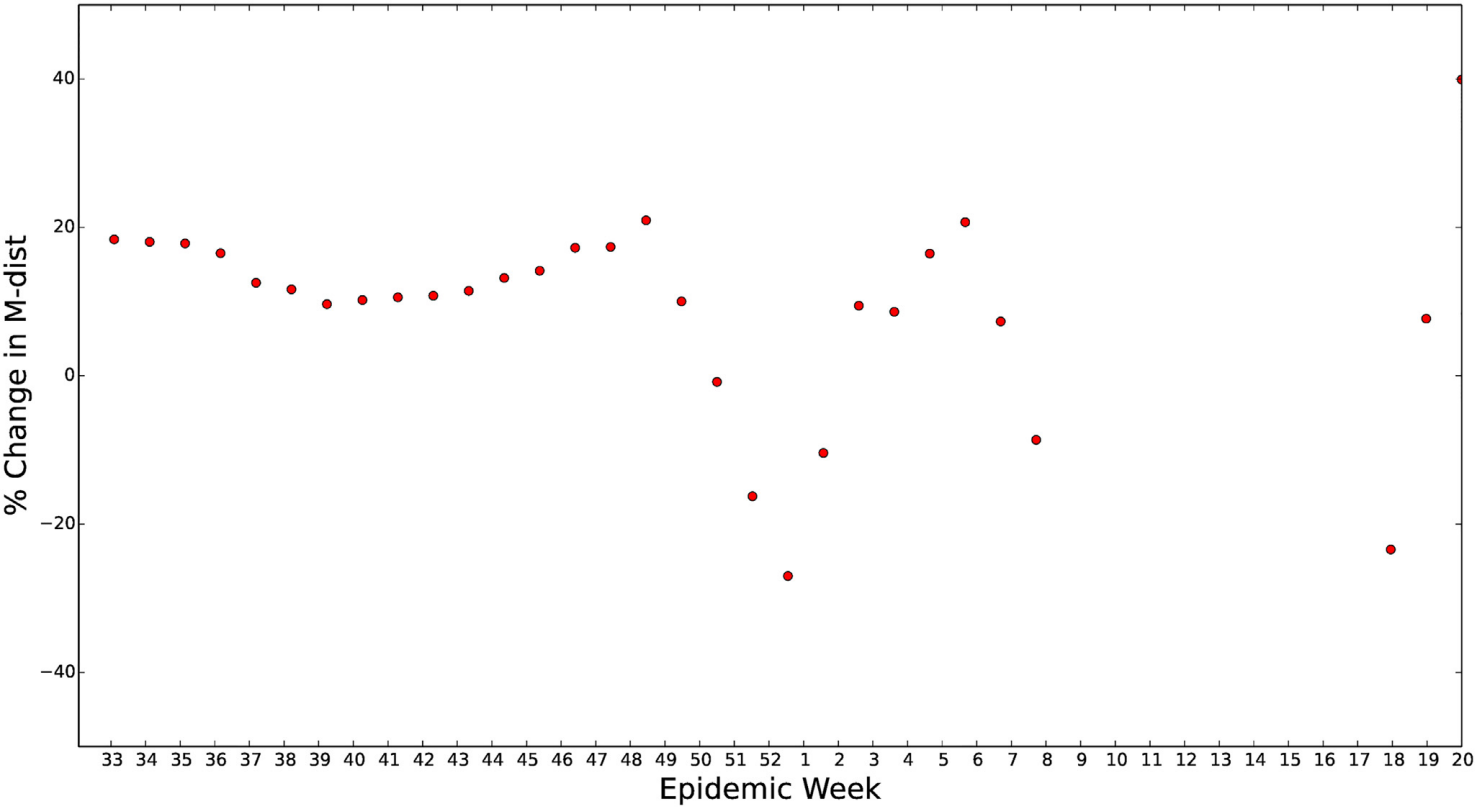
Percent improvement in M-distance using *S* ^*ν*^ *EIR* with enKS to forecast 2013–2014 U.S. ILI data. The percent improvement in the M-distance for the U.S. 2013–2014 ILI forecast using the data assimilative method compared to the straw man method is shown. Here we see that up to a month before the peak of ILI the use of a mathematical influenza model with data assimilation provides up to a 20% improvement in the forecast with a minimum of a 10% improvement. However, this improvement is quickly degraded due to model bias close to the peak. It is notable that after the peak is observed the data assimilation attempts to correct the model but can not make up for the *S* ^*ν*^ *EIR* model’s strong tendency to a zero infected state after the peak.

In the early part of the influenza season, the improvement of the data-assimilative forecast over the straw man forecast is more apparent if we look at the percent improvement in the straw man’s M-distance that the data assimilative M-distance represents, Fig 14. Up until a week or two before the peak of 2013–2014 ILI the use of the *S* ^*ν*^ *EIR* model together with the enKS data assimilation scheme offers up to a 20% improvement in the M-distance of the straw man forecast. Since the majority of public health decisions are made well before the peak of ILI this represents a significant advantage of the data-assimilative method over the straw man method. We are confident that with further improvements to the prior and the model this improvement can be increased.

#### Qualitative accuracy

Each week, given the 300 infected time series from the analyzed ensemble, we gain 300 samples of the epidemic peak percent infected, the epidemic start time, the epidemic duration, and the week of the epidemic peak. From these 300 samples of these quantities of interest, we estimate the 5%, 25%, 50%, 75%, and 95% posterior quantiles. This gives us a convenient weekly summary of our influenza forecast with uncertainty quantified by 50% and 90% posterior credible intervals about the median. It is important to clarify that, since we are adjusting the underlying parameterization and initialization of the *S* ^*ν*^ *EIR* model and not the state of the model directly, the median and credible intervals for quantities such as the start week of the elevated ILI season continue to be adjusted even after they are observed. It is important to report these adjustments as they show how later observations effect the model’s dynamics throughout the entire season. For example, we can observe that the enKS applied to the *S* ^*ν*^ *EIR* model in the later ILI season tries to maintain an elevated ILI level by pushing the simulated peak forward in time. In practical application, the *forecast* of quantities such as the start week would be fixed once they were observed.

**Fig 15:**
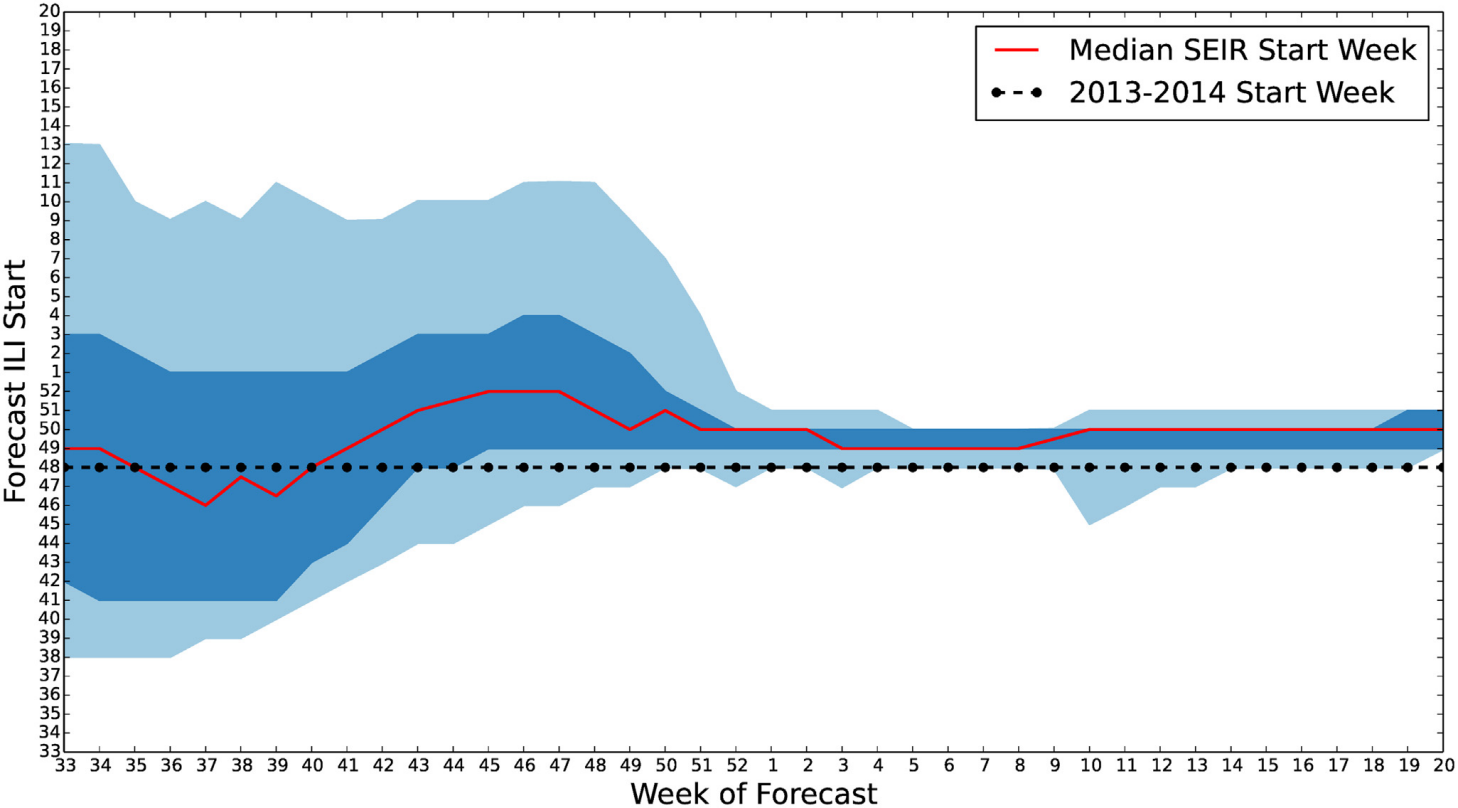
*S* ^*ν*^ *EIR* start week quantiles for 2013–2014 U.S. ILI. 50% and 90% credible interval estimates of the influenza season start week are plotted along with the median. Each week, as new ILI data become available the forecast is revised. This causes the uncertainty in our forecast to diminish. However, due to the model’s inability to maintain an elevated ILI level past the peak, we see that late in the flu season, the model adjusts by pushing the start week later into the season. This causes an overestimation of the start week that worsens as the season progresses. In practice, once the start week has been observed the *forecast* would be fixed. However, adjustment of the model parameterization using the enKS would continue to affect the model simulation start date.

A time series plot of the start week credible intervals for our seasonal *S* ^*ν*^ *EIR* forecast is shown in Fig 15. A similar plot for the start week credible intervals forecast using the straw man model would show a constant distribution with median start week forecast at the 38^th^ epidemiological week. We can see for Fig 15 that the high probability region for start week, as forecast by our epidemiological model, is usually 1–2 weeks after the actual 2013–2014 start week. However, the actual start week is contained within the 90% credible region until a week or two after the peak of flu season. This region is also seen to constrict as ILI and Wikipedia observations are assimilated.

**Fig 16:**
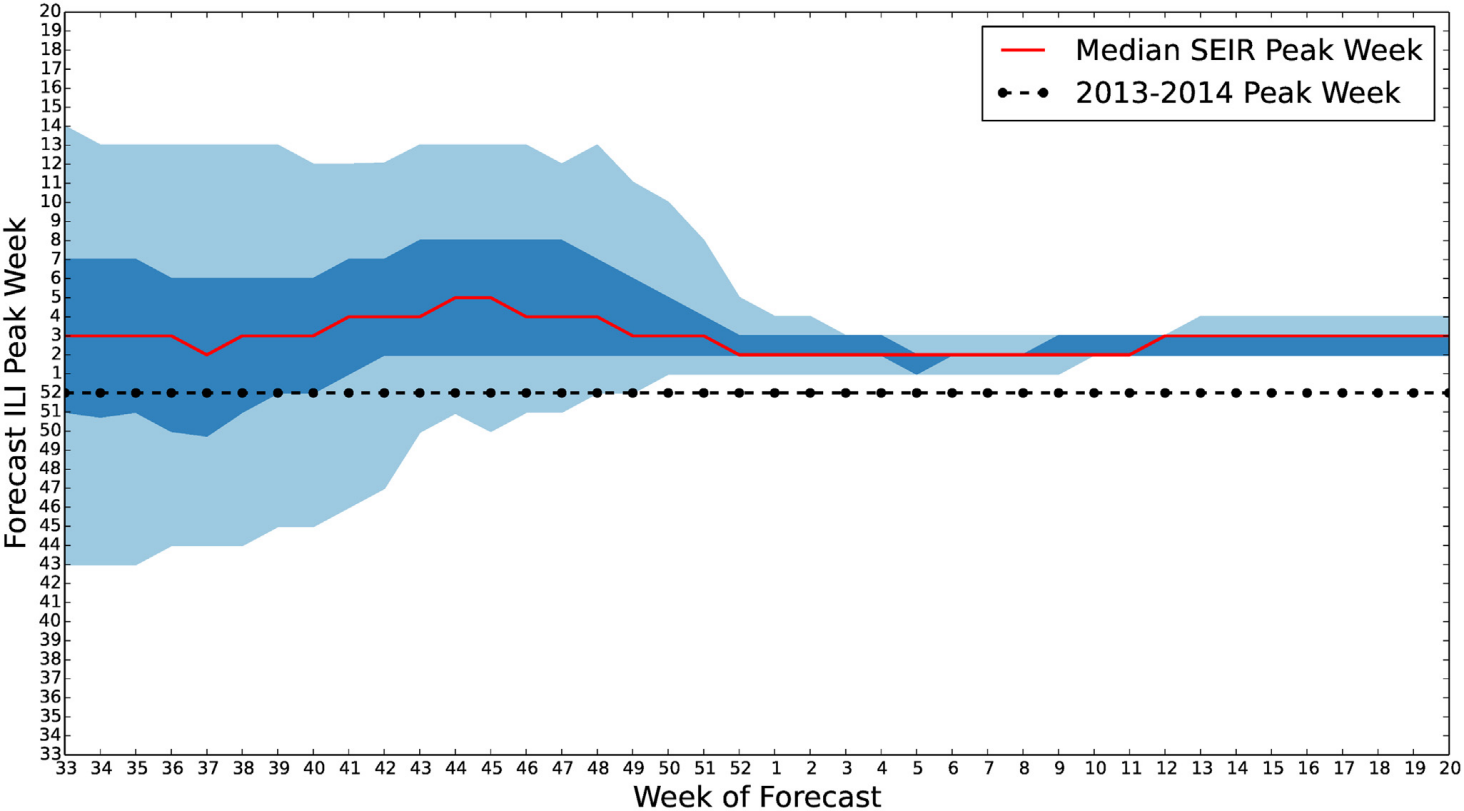
*S* ^*ν*^ *EIR* peak week quantiles for 2013–2014 U.S. ILI. 50% and 90% credible interval estimates of the influenza season peak week are plotted along with the median. Due to our prior and biases in our model the forecast for the week of ILI peak is consistently later than the observed 2013–2014 peak. However, we can see here that, as ILI observations are assimilated, early peaks are eliminated from the forecast prior to observation of the peak.

**Fig 17:**
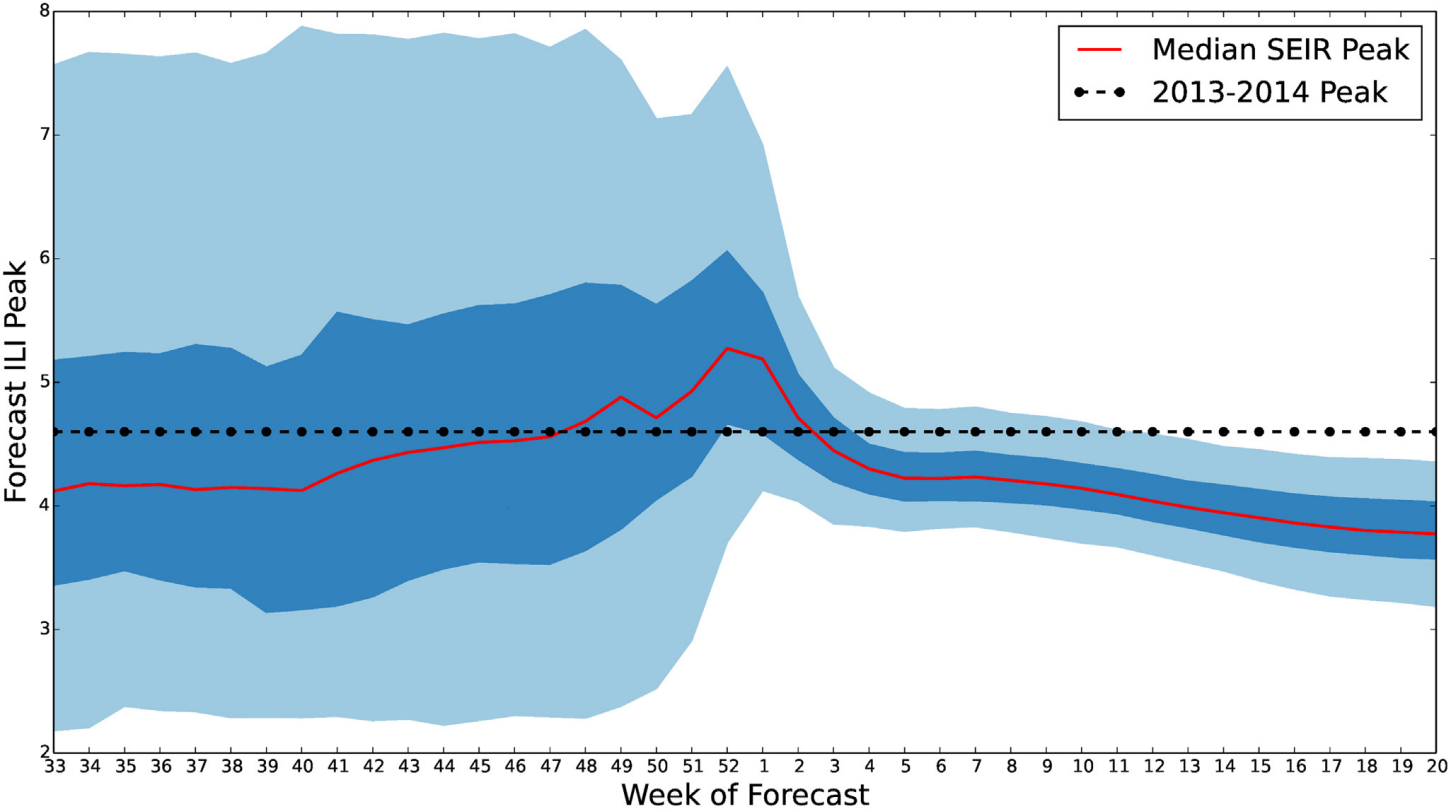
*S* ^*ν*^ *EIR* peak quantiles for 2013–2014 U.S. ILI. 50% and 90% credible interval estimates of the influenza season peak are plotted along with the median. Forecasts for the size of the ILI peak were widely varying in the 90% credible interval. This could possibly be reduced by the elimination of high peak outliers such as the 2009 H1N1 emergence and through adjustment of low forecasts in our prior. However, even with these draw backs the 50% credible region has a width of only 1%–2%.

We calculated similar time series of credible intervals for the forecast peak week (Fig 16), and the magnitude of the ILI peak (Fig 17). The forecast for the ILI peak was uncertain with the 90% credible interval having width of around 6% until close to the actual peak. However, the median forecast for the peak week was consistently within one or two weeks of the actual observed peak. These results suggest that our model and prior were calibrated to predict the timing of the start and peak of influenza season well but underestimate the size of the peak.

When compared to the forecast start week from the straw man model, our *S* ^*ν*^ *EIR* model seems like a favorable tool. The straw man’s forecast does not assimilate current observations and thus its predicted start week is constant. Two things should be mentioned about calculating these credible intervals for the straw man forecast. First, since a sample from the straw man forecast has no week-to-week correlations, the weekly forecasts can vary greatly from week to week. This is a problem when computing the start week for the influenza season since a given straw man sample does not remain above 2% for consecutive weeks. Second, for similar reasons the duration cannot even be defined for one time series sample of the straw man forecast. The lack of correlations in week-by-week forecasts in the straw man model severely restricts its usefulness in influencing public health policy. For example, even after the 2013–2014 ILI data had been observed to decrease over the course of five weeks the straw man’s median prediction was increasing. This makes it difficult to use to interpret the dynamics of influenza progression. On the other hand, even though the *S* ^*ν*^ *EIR* model has significant errors in the forecast after the peak the trend of decreasing ILI is in agreement.

## Discussion

### Summary of method

We have outlined an approach to forecasting seasonal influenza that relies on modern ensemble data assimilation methods for updating a prior distribution of a disease transmission model. The method used a dynamic compartmental model of influenza spread that has been used in previous research [51, 52, 54] but is applicable to any compartmental model of disease with a regularly updated public health data source. Though the flu forecasting system we have presented here is not ready to entirely base public health policy on, the methods provide a valuable framework. In particular, this framework provides real-time model testing, model dependent prior estimation, evaluation of probabilistic disease forecasting, and transitioning from a deterministic model to a probabilistic forecast. Moreover, if prior to the peak our model predicts an unexpected change in the future number of cases, then this is an indication to public health decision makers that the model may be picking up on hidden events and trends that are being missed by more traditional statistical prediction methods. In this regard, the model forecast is similar to having another expert viewpoint available during the decision process that can identify trends that might have otherwise been missed.

We evaluated the accuracy of our forecast using the M-distance, based on the Gaussian likelihood of observations, and the deviation of time series of quantiles for a set of quantities of interest arising from flu dynamics. Both of these methods were used on our data assimilation approach and on a much simpler forecast using estimated normal distributions. The application of these measures of accuracy, combined with our specific approach to data assimilation with a dynamic model of the influenza dynamics allowed us to highlight model inaccuracies that can then be improved in the future.

Though a statistically simple tool, the inclusion of a straw man forecast as a baseline to evaluate our data assimilation scheme’s usefulness is indispensable when evaluating measures of accuracy. Especially for some measures, such as the M-distance, it is difficult to tell whether or not a value implies the forecast performed well without a baseline. We hope that the approach of measuring a forecast against a baseline becomes established practice in future developments of epidemic forecasting.

The differential equation representation of influenza dynamics, modeled proportions of the population as susceptible, exposed/non-infectious, symptomatic/infectious, and recovered/immune/removed. The model did not allow for any re-infection of influenza, which is thought to be biologically accurate for at least a single strain of flu in a single season [56]. We also modeled the effect of heterogeneity in the influenza contact network and seasonal variation in the transmissibility of flu. Our method of data assimilation adjusted the allowable parameterizations and initializations of this model as ILI data became available.

The forecast was made up of actual realizations of our *S* ^*ν*^ *EIR* model used. This has the arguable advantage of highlighting observed sections of the ILI data stream that differ drastically from the model’s assumptions. However, since the model state is not adjusted at each ILI data point directly, the forecast with an incorrect model eventually diverges from the data.

To iteratively update the prior distributions of parameterizations and initializations, we used an ensemble Kalman smoother. This was observed to significantly pull the model toward a subset of parameterizations and initializations that agreed well with the data. Since the model seemed to be reasonably adjusted toward observations and retained a significant amount of ensemble variation in the forecast, there is strong evidence that the assimilation scheme works well. The challenge now is to arrive at a model that more accurately represents influenza dynamics perhaps by including considerations made in [53, 55–59].

Our quantitative measure of forecast accuracy is motivated by the Gaussian likelihood function and has been used, in many instances, to assign a value to the distance from some predicted distribution with a fixed mean and covariance. This is exactly the setting we are in, when we make the Gaussian assumptions inherent in the Kalman filter methods. The application of the M-distance in this instance showed that our model performed better than the simple straw man forecast in the beginning of the season but then systematically diverged from the late season ILI data.

Besides demonstrating the accuracy of our forecast at capturing overall dynamics, we also quantified our forecating method’s ability to accurately estimate quantities of interest relating to the impact of a given influenza season. We showed how the time series of forecast median and posterior credible intervals for the season’s start week, peak week, duration, and peak level changed over time. This measure in particular demonstrated the advantages of having an underlying mechanistic model as compared to the purely statistical normal approximation forecast as used in the straw man forecast.

### Future improvements and lessons learned

This work shows the viability of using a data assimilation method to sequentially tune a model of disease dynamics. However, it also highlights the need to use caution when adjusting the model to match data. If balances inherent in the model are not maintained during each adjustment step, it is possible to forecast data accurately with a model that is incorrect (e.g., one that has no single realization that will reproduce the data up to data error). The upside of this approach is that if only the model parameterization and initialization are adjusted, this type of forecasting process allows one to identify the assumptions of the model that diverge from observations. This is an important tool, to advance models to more accurate representations of reality, that could be ignored if data assimilation methods are used to adjust a model’s state and parameterization throughout the forecast.

The method proposed here, which maintain *S* ^*ν*^ *EIR* balances during assimilation, are not the only possible methods of maintaining the population balances assumed in a compartmental disease model. More research needs to be done on the best way to adjust a model to observations while maintaining an accurate representation of disease model balances. Moreover, if the goal is to create forecasts for multiple seasons, forecasting from initial conditions will not always be viable. It remains an important open research question as to how far in the past one should optimally start forecasts from. The farther in the past a forecast is made from, the more dynamics of the model and data are assimilated. The downside of this is that considering too much of the models dynamics can impose unnecessary restrictions on the prior, leading to a divergent forecast.

A major concern for our epidemiological model is the systematic divergence from the data at the end of the influenza season. This divergence is evident in the optimal fitting done with our *S* ^*ν*^ *EIR* model on historical ILI data. Since we did not know, before completing this work, which factors in disease forecasting would be most important, we have only added complexity in the model to account for some heterogeneity in the contact network of influenza spread and seasonally-varying transmissibility of influenza. Spatial spread of influenza does have a good data source that could be used in the future since ILI is collected in 10 different Health and Human Services regions for the United States. However, a disease model that links spatial spread in each of these regions would have significantly more parameters to determine in a prior distribution. Moreover, the level of ILI error between regions seems to be highly variable when observing historical ILI time series from different regions. The variability between regional ILI reporting methods poses a challenge beyond the scope of this first approach.

Behavior change was not incorporated into this work since regularly updated observations of human behavior changes affecting influenza spread are, to the authors knowledge, not available. Vaccination data are available but are not updated on a time scale fine enough to be comparable with weekly ILI. Moreover, vaccination rates would directly reduce the proportion of the population susceptible to influenza. Unfortunately, only sparse data are available on the actual proportion of the population susceptible to a given influenza strain. Thus, incorporation of vaccination rates into a forecast is not obvious until there exists a good method to directly determine the proportion of susceptibles in the United States.

We did not include multiple strains in our forecasts since it was not obvious to us that a single strain model would fail. This belief was based on the fact that a single strain model, with the inclusion of variable transmission, has surprisingly flexible dynamics and thus may be able to fit ILI dynamics well even without biological correctness. Secondly, the introduction of multiple strains into a model adds many more parameters to the model, making it difficult to determine a prior distribution from historical data and increasing the problem of “lack of determination” in the model. However, there are regularly updated data on prevalence of specific strains provided by the WHO/NREVSS [6] and therefore, in retrospect, inclusion of multiple strains may be highly advantageous in the future. These data show that often there are one or two outbreaks of secondary dominant influenza strains in the late season. We hypothesize that these secondary strains are a primary cause of the heightened tail in the ILI data and we will investigate a multi-strain influenza model [56] in future forecasting work.
